# Niacin inhibits vascular calcification via modulating of SIRT1/SIRT6 signaling pathway

**DOI:** 10.1038/s41420-025-02882-2

**Published:** 2025-12-06

**Authors:** Chao-hua Kong, Li-da Wu, Yue Sun, Xiao-min Jiang, Yi Shi, Feng Wang, Dong-chen Wang, Yue Gu, Wen-ying Zhou, Jin-que Luo, Shao-liang Chen, Yue-lin Chao

**Affiliations:** 1 Department of Cardiology, Nanjing First Hospital, Nanjing Medical University, Nanjing, China; 2 Hunan Provincial Key Laboratory of the Research and Development of Novel Pharmaceutical Preparations, “The 14th Five-Year Plan” Application Characteristic Discipline of Hunan Province (Pharmaceutical Science), College of Pharmacy, Changsha Medical University, Changsha, Hunan China

**Keywords:** Calcification, Cell signalling

## Abstract

Vascular calcification (VC) is a common pathological state that often accompanies calcium-phosphorus metabolism disorder and chronic kidney diseases (CKDs). Vascular smooth muscle cell (VSMC) has been widely acknowledged as one of the main cell types involved in this process. Niacin, a lipid-lowering reagent, has been demonstrated to be beneficial in atherosclerotic disease, but its role in vascular calcification remains unexplored. Restricted cubic spline (RCS) analysis of clinical datasets revealed an inverse correlation between dietary niacin intake and abdominal aortic calcification (AAC). Our data showed that niacin treatment remarkably reduced VSMC osteogenic differentiation. Moreover, niacin treatment alleviated CKD and vitamin D_3_-induced vascular calcification in C57BL/6J mice. Mechanistically, we for the first time demonstrated that niacin inhibited vascular calcification via maintaining both Sirtuin 1 (SIRT1) and Sirtuin 6 (SIRT6) levels. Further, we verified that niacin increased SIRT1 and SIRT6-mediated autophagy flux in VSMC. Our findings reveal that niacin exerts anti-calcification effect via maintaining both SIRT1 and SIRT6, providing novel therapeutic strategies in the treatment of vascular calcification.

## Introduction

Vascular calcification is characterized by deposition of calcium-phosphate complexes in the tunica media and is considered as a degradative process associated with natural aging as well as pathological conditions such as diabetes and chronic kidney diseases (CKDs) [1–4]. The vasculature system is the second most calcified tissue after the skeleton and many studies have suggested that vascular calcification is a gene-related transdifferentiation process resembling bone mineralization [5]. Accumulating evidence have convinced that osteoblast-like dedifferentiation of VSMC plays the most essential pathological role in vascular calcification. Targeting the osteogenic transition of VSMC becomes an important and promising strategy in the prevention and treatment of vascular calcification.

Mechanisms associated with vascular calcification including inflammation, cellular autophagy, endoplasmic reticulum stress, cellular senescence, and aberrant calcium-phosphate metabolism [6–9]. Of note, vascular aging constitutes the fundamental pathophysiological basis for various cardiovascular diseases, including atherosclerosis, vascular calcification, and aneurysm [10]. Senescent VSMC acquires an elongated morphology and exhibits reduced contractility, and are prone to accelerated osteogenic transition upon pathological stimulation [11]. The Sirtuin family comprises 7 members and plays an important role in regulating cellular longevity [12]. Previous studies have indicated SIRT1 and SIRT6 are involved in vascular calcification [13, 14]. Both SIRT1 and SIRT6 are nicotinamide adenine dinucleotide-dependent histone deacetylases that involved in regulating cell proliferation, differentiation, and apoptosis [15–17]. Agents that preserve SIRT1 and SIRT6 expression may attenuate vascular calcification [18].

Nicotinic acid (Niacin) serves as an alternative lipid-lowering agent for statin-intolerant patients and has been shown to reduce mortality in patients with atherosclerosis [19]. Niacin treatment is reported to be protective for cardiovascular diseases by mechanisms such as reducing monocyte/macrophage inflammatory responses, regulating oxidative stress, and alleviating hyperlipidemia [20–22]. In vivo, niacin converts to nicotinamide adenine dinucleotide (NAD^+^), an essential cofactor for numerous multifunctional enzymes, including SIRT1 and SIRT6 [23–25]. Recently, niacin supplement is found to attenuate pulmonary artery hypertension and abdominal aortic aneurysm [26–28]. However, whether niacin can abrogate vascular calcification remains unknown.

In this study, we evaluated whether niacin treatment counteracted arterial calcification in both CKD and VD3-induced vascular calcification mouse models. Our results demonstrate that niacin mitigates vascular calcification by upregulating SIRT1 and SIRT6 expression and activating VSMC autophagy. These findings provide new insights in the prevention and treatment of vascular calcification.

## Results

### Niacin intake is negatively correlated with abdominal artery calcification

**Fig. 1:**
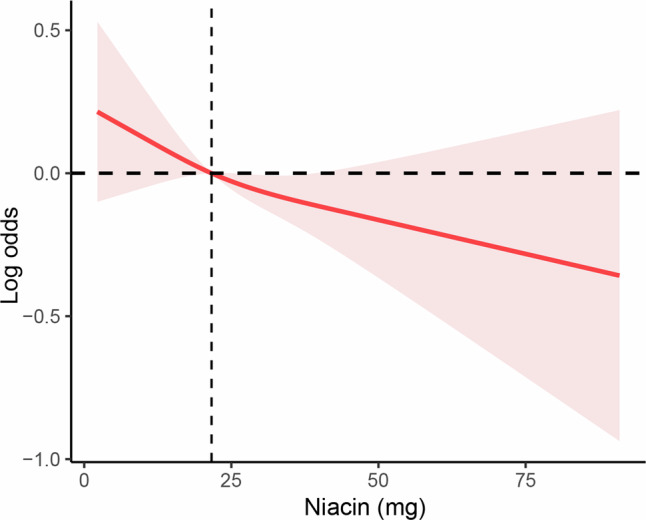
RCS analysis of the association between niacin intake and AAC. RCS analysis showed that niacin intake is negatively correlated with AAC incidence.

In this study, we included 2897 participants, of which 867 had abdominal artery calcification (AAC) and 2030 did not. As shown in Supplementary Table S1, baseline data indicated that participants in the AAC group were older than those in the non-AAC group. There was no significant difference in gender composition between the AAC and non-AAC groups. We found that the proportion of participants with an annual household income of less than $2000 was significantly higher in the AAC group compared to the non-AAC group. Additionally, the proportion of obese and overweight participants was significantly higher in the AAC group. Notably, the proportion of smokers was significantly higher in the non-AAC group, possibly due to the younger age of participants in this group. However, there was no significant difference in the proportion of drinkers between the two groups. Given that AAC and systemic arterial stiffness and calcification can lead to elevated blood pressure, the proportion of participants with hypertension was significantly higher in the AAC group. Since AAC can be partly due to abnormal calcium and phosphorus metabolism, we found that eGFR was higher in the AAC group compared to the non-AAC group. Furthermore, there were significant differences in lipid profiles between the two groups, as detailed in Supplementary Table S1. After adjusting for age, sex, race, household income, education level, obesity, smoking, drinking, hypertension, eGFR, blood calcium, and phosphorus levels, we further explored the relationship between dietary niacin intake and the risk of AAC. Using the RCS curve method, we performed a linear fit of this relationship. Our results suggested that as niacin intake increased, the risk of AAC significantly decreased, showing a linear negative correlation between the two (Fig. 1).

### Niacin attenuates vascular smooth muscle cell calcification

**Fig. 2:**
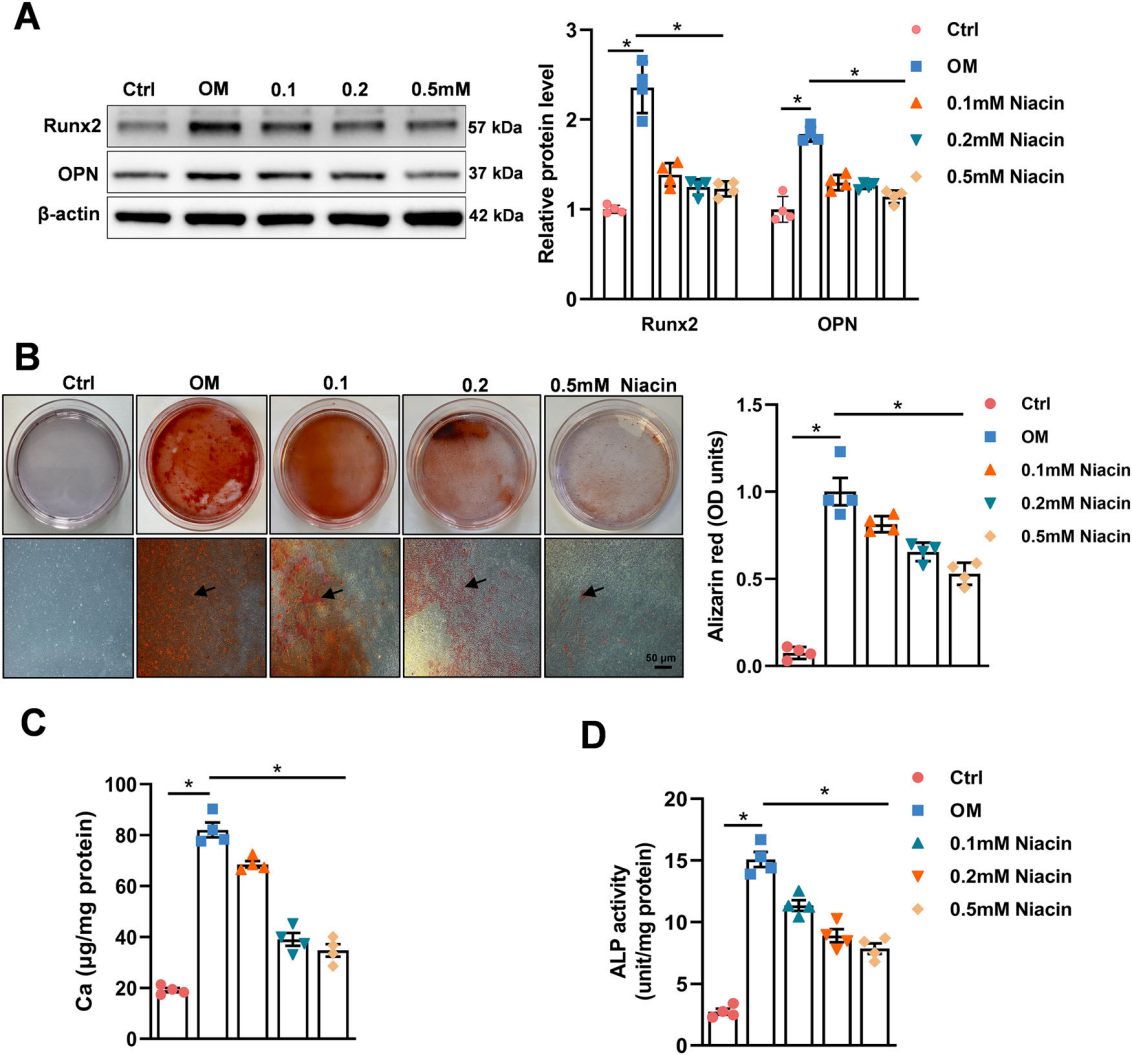
Niacin inhibits rat aortic vascular smooth muscle cell calcification. Rat vascular smooth muscle cells (RASMCs) were incubated with growth medium (Ctrl), or osteogenic medium (OM) supplemented with or without niacin with different concentration (0.1, 0.2, and 0.5 mM) (*n* = 4). **A** Western blot was used to examine the expression levels of Runx2 and OPN after stimulation for 3 days. **B** Representative images showing cells stained with Alizarin red solution at day 14. Scale bar = 50 μm. **C** Quantitative analysis of calcium content using a Ca assay kit. **D** Quantitative analysis of ALP activity of RASMCs. * *P* < 0.05, ** *P* < 0.01. One-way ANOVA followed by Tukey’s post hoc test.

To explore whether niacin treatment affects VSMC calcification. Primary rat aortic smooth muscle cells (RASMCs) were cultured in osteogenic medium (OM) containing high phosphate (2.6 mmol/L) to induce calcification. 0.1, 0.2, or 0.5 mmol/L niacin was added in calcified medium [29]. Of note, OM-cultured VSMC exhibited distinct osteogenic phenotype as evidenced by significant upregulation of osteogenic markers runt-related transcription factor 2 (Runx2) and osteopontin (OPN) (Fig. 2A) and marked increase of Alizarin red S staining (Fig. 2B). Intriguingly, the expression of osteogenic markers was remarkably downregulated in niacin-treated VSMC compared with the untreated controls in a dose-dependent manner (Fig. 2A). Niacin treatment significantly alleviated OM-induced VSMC calcification at day 14 as confirmed by qualification analysis of Alizarin red S staining (Fig. 2B). Consistently, calcium content assay and alkaline phosphatase (ALP) activity assay showed that niacin treatment reduced both calcium content (Fig. 2C) and ALP activity (Fig. 2D).

### Niacin inhibits aortic calcification in vivo

**Fig. 3:**
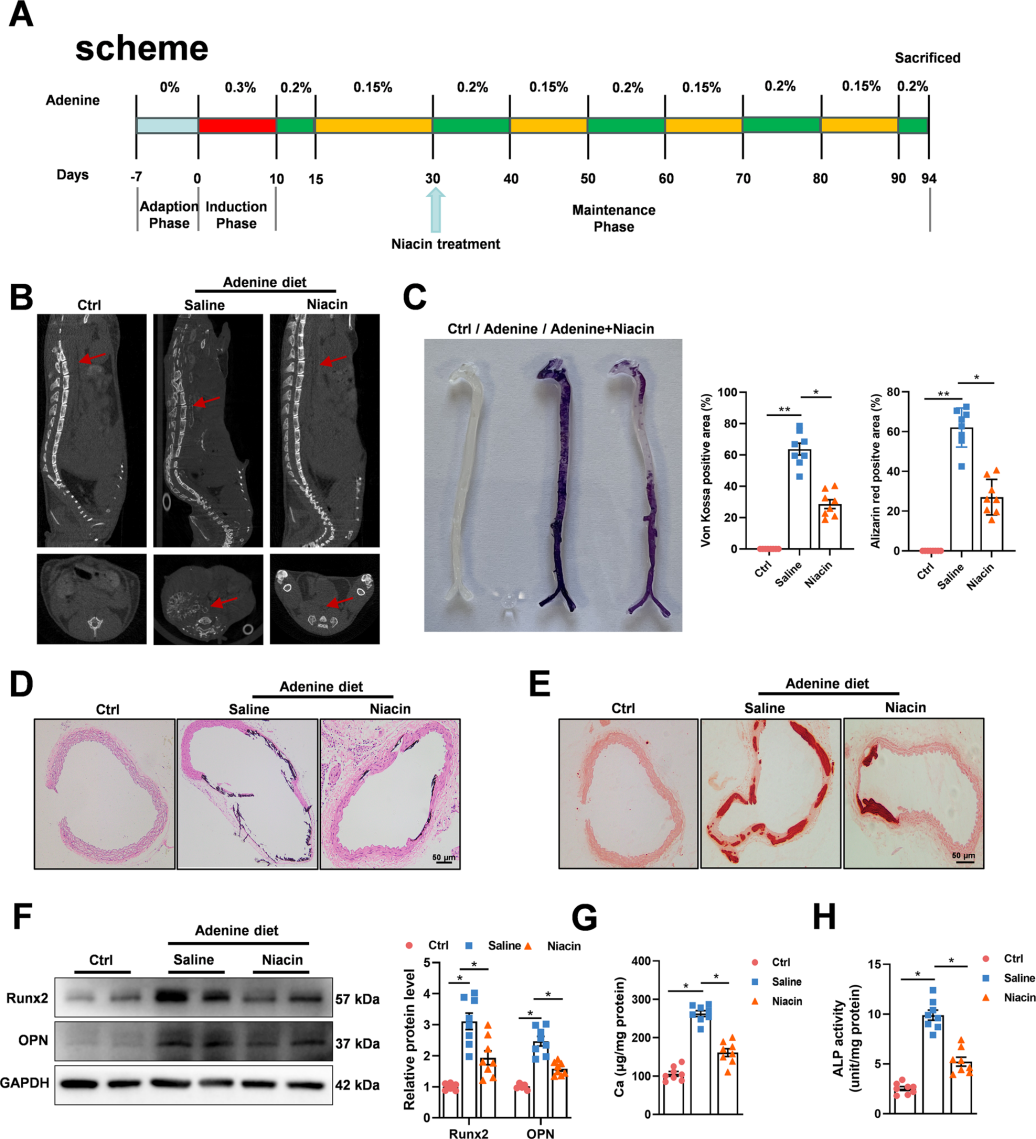
Niacin supplement alleviates CKD-induced vascular calcification in C57BL/6J mice. Adenine diet-induced CKD mice were treated with niacin (600 mg/kg) via gavage for 9 weeks (*n* = 8). **A** Scheme of the construction of the CKD-associated vascular calcification model and niacin supplement. **B** Aortic calcification (red arrow) was examined by micro-CT. **C** Mineral deposition was detected by Alizarin red staining of whole mount of aorta. Representative images showing aortic arteries staining with Alizarin red. **D** and **E** Representative images of Alizarin red and von Kossa staining of aortic sections. Scale bar = 50 μm. **F** Representative western blot analysis and quantification of Runx2 and OPN protein levels in arteries from different groups. **G** Calcium content of aortas was measured. **H** Quantification of ALP activity. Data are shown as mean **±** SEM. * *P* < 0.05, ** *P* < 0.01. One-way ANOVA, Tukey’s HSD post hoc test.

To further evaluate niacin’s effect on CKD-induced vascular calcification in vivo. C57BL/6J mice were randomly assigned to control diet, adenine diet (AD), and AD + niacin group. An AD-induced CKD model was established as previously described (Fig. 3A) [30]. CKD mice showed reduced body weight and elevated serum levels of blood urea nitrogen, creatinine, and phosphate compared to the control group. However, no significant differences in serum calcium, creatinine, or phosphate levels were observed between the AD and AD + N groups before week 12 (Supplementary Table S2). We next performed micro-CT to assess the extent of aortic calcification. Micro-CT analysis indicated that niacin-treated mice exhibited less calcification areas compared with saline-treated mice (Fig. 3B). Alizarin red S staining of whole aortas showed niacin supplementation attenuated CKD-induced aortic calcification (Fig. 3C). As anticipated, von Kossa (Fig. 3D) and Alizarin red S staining (Fig. 3E) of aortic sections demonstrated reduced vascular calcification after niacin treatment. Immunoblotting results further demonstrated decreased expression of calcifying markers in aortic arteries from the niacin group (Fig. 3F). In addition, niacin treatment significantly reduced calcium content in the aortic arteries (Fig. 3G) and serum ALP activity (Fig. 3H). Collectively, these results showed that niacin supplementation attenuated CKD-induced vascular calcification in mice.

Next, we used VD_3_ injection-induced vascular calcification model to address the limitation of single model (Supplementary Fig. S1A). No significant alteration in body weight or serum mineral metabolites was observed between groups (Supplementary Table S4). Similarly, we observed less calcification areas in niacin group by Alizarin red S staining of whole aortas (Supplementary Fig. S1B). Von Kossa (Supplementary Fig. S1C) and Alizarin red S staining (Supplementary Fig. S1D) of aortic sections showed niacin treatment alleviated VD_3_ overload-induced vascular calcification. Moreover, immunoblotting results indicated niacin treatment downregulated osteogenic markers in calcified aortic arteries (Supplementary Fig. S1E). Taken together, the above data demonstrated that niacin treatment mitigated medial arterial calcification in vivo.

### SIRT1 and SIRT6 are involved in niacin inhibited VSMC calcification

**Fig. 4:**
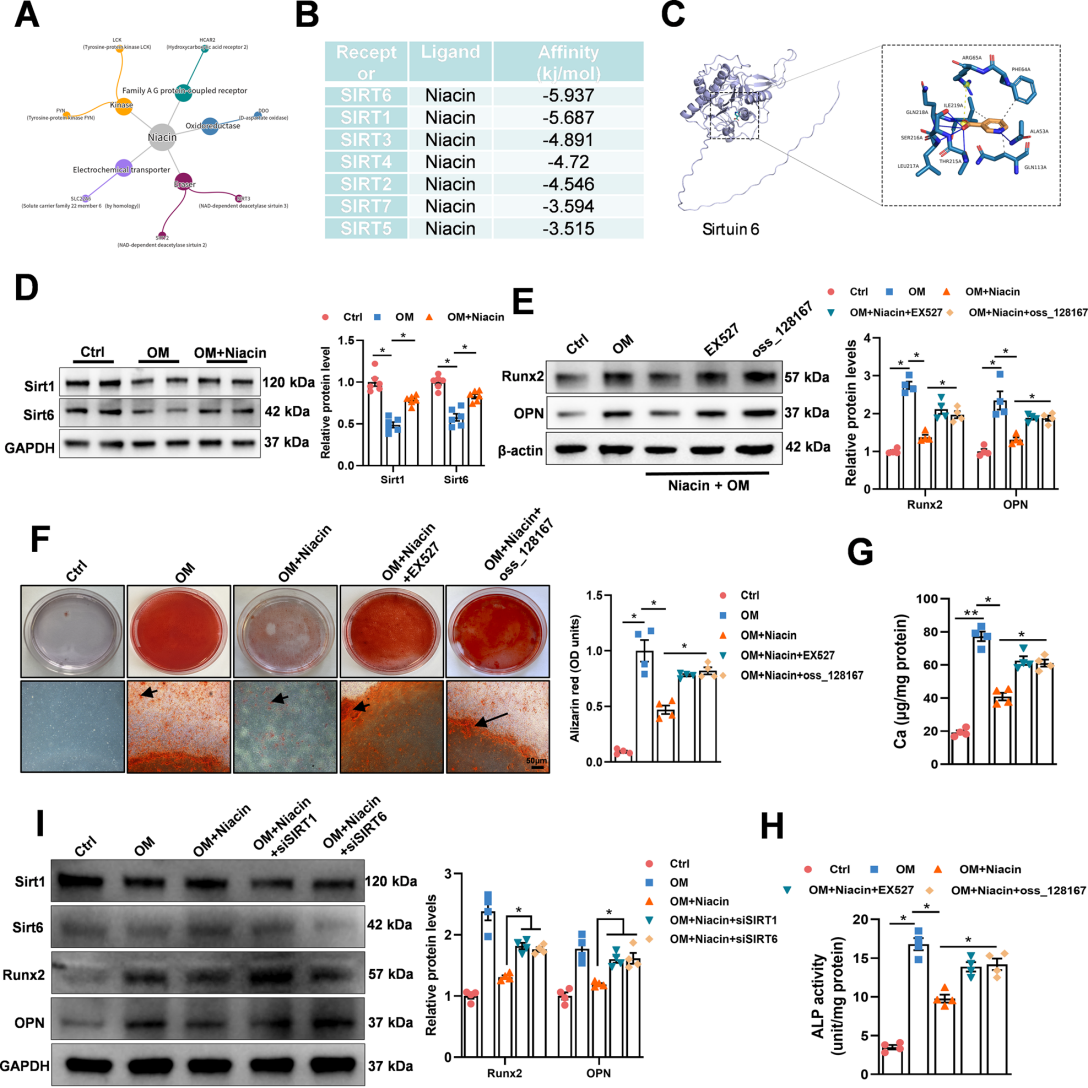
Niacin inhibited vascular calcification associated with SIRT1 and SIRT6 signaling. **A** SwissTargetPrediction tool was used to predict niacin-binding proteins. **B** Molecular docking results showing the confidence of 7 Sirtuins binding to niacin. **C** Molecular interacting pattern between niacin and SIRT6. **D** Representative images and quantification of western blots for the expression levels of SIRT1 and SIRT6. **E** RASMCs were treated with SIRT1 inhibitor EX527 or SIRT6 inhibitor OSS_128167. Representative western blots for the protein expression of Runx2 and OPN. **F** Representative images showing cells stained with Alizarin red solution at day 14. Scale bar = 50 μm. **G** and **H** Quantitative analysis of calcium content or ALP activity. **I** RASMCs were transfected with SIRT1 and SIRT6 siRNA in the presence of niacin and OM, representative images of western blots for the expression of Runx2 and OPN are shown. Data are shown as mean **±** SEM. * *P* < 0.05, ** *P* < 0.01. One-way ANOVA, Tukey’s HSD post hoc test.

To verify niacin’s inhibitory effects on vascular calcification. We performed RNA sequencing on arteries from AD and AD + niacin groups (*n* = 3 for each group). Whole transcriptome analysis identified 1834 upregulated and 1043 downregulated genes (fold change >2, *P* < 0.05) (Supplementary Fig. S2A). Kyoto Encyclopedia of Genes and Genomes analysis showed that downregulated genes in niacin group were enriched in pathways associated with bone mineralization and calcium-binding, consistent with our previous results (Supplementary Fig. S2B). Gene set enrichment analysis validated the reduced expression of calcification (Supplementary Fig. S2C) and cellular senescence (Supplementary Fig. S2D) associated genes in niacin-treated vessels. Upregulated genes were enriched in nucleotide metabolism (Supplementary Fig. S2E) and longevity-regulating (Supplementary Fig. S2F) pathway. Further, to find direct binding target of niacin, SwissTargetPrediction tool was used. Among 5 potential protein family candidates, the cellular longevity pathway highly correlated Sirtuin family was identified (Fig. 4A). Subsequent molecular docking analysis of niacin with all seven Sirtuin members revealed high binding affinity for SIRT1 and SIRT6 (Fig. 4B, C). Thus, we examined if niacin regulated SIRT1 and SIRT6 under calcifying condition. Immunoblotting revealed that niacin treatment partially reversed OM-induced downregulation of SIRT1 and SIRT6 in VSMC (Fig. 4D). To determine the functional role of SIRT1/SIRT6 signaling in niacin-mediated inhibition of VSMC calcification. We incubated RASMCs with SIRT1 inhibitor (EX527) or SIRT6 inhibitor (OSS_128167). Immunoblotting result showed that EX527 or OSS_128167 treatment diminished niacin-induced downregulation of Runx2 and OPN (Fig. 4E). In addition, Alizarin red S staining indicated that either EX527 or OSS_128167 incubation partially blocked niacin-inhibited VSMC calcification (Fig. 4F). Moreover, both inhibitors reversed niacin-induced reductions in calcium deposition (Fig. 4G) and ALP activity (Fig. 4H).

To validate SIRT1/SIRT6 signaling in niacin’s anti-calcification mechanism, we performed RNA interference to knockdown SIRT1 or SIRT6 in VSMC. Western blotting confirmed knockdown efficiency (Supplementary Fig. S3A, B). Immunoblotting revealed that SIRT1 or SIRT6 silencing abrogated niacin-induced downregulation of RUNX2 and OPN (Fig. 4I). These data suggested that the SIRT1 and SIRT6 signaling are critical in niacin-inhibited VSMC calcification.

### Niacin attenuated aortic calcification in CKD mice via SIRT1 and SIRT6 signaling

**Fig. 5:**
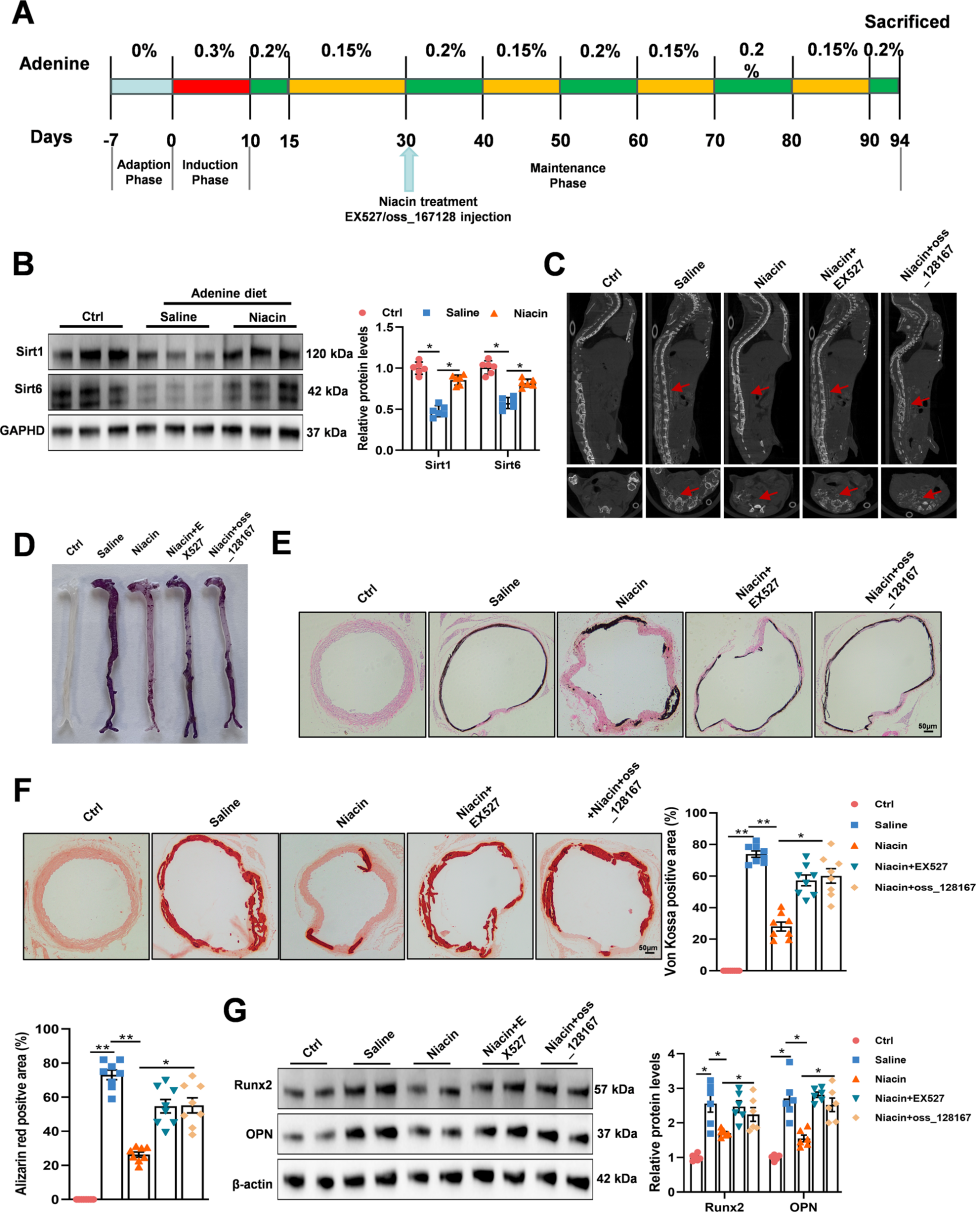
Effects of SIRT1 inhibitor and SIRT6 inhibitor on aortic calcification in CKD mice. Adenine diet-induced CKD mice were treated with niacin (600 mg/kg) together with EX527 (5 mg/kg/d, i.p.) or OSS_128167 (10 mg/kg/d, i.p.) for 9 weeks (*n* = 8 for each group). **A** Scheme of the construction of the CKD-associated vascular calcification model and EX527 or OSS_128167 supplement. **B** Western blots for the expression of SIRT1 and SIRT6 of aortic arteries. **C** Representative images of aortic calcification (red arrows) are shown after examining by micro-CT. **D** Mineral deposition was detected by alizarin red staining of whole mount of aorta. Representative images showing aortic arteries staining with Alizarin red. **E** and **F** Representative images of von Kossa and Alizarin red staining of aortic sections. Scale bar = 50 μm. **G** Representative western blot analysis and quantification of Runx2 and OPN protein levels in arteries from different groups. Data are shown as mean **±** SEM. * *P* < 0.05, ** *P* < 0.01. One-way ANOVA, Tukey’s HSD post hoc test.

To validate the requirement of SIRT1 and SIRT6 pathway in niacin-mediated attenuation of vascular calcification in vivo. We administered EX527 or OSS_128167 to CKD mice supplemented with niacin (Fig. 5A). Immunoblotting showed that niacin treatment reversed CKD-induced SIRT1 and SIRT6 downregulation in calcified vascular (Fig. 5B). Micro-CT analysis revealed that EX527 or OSS_128167 administration exacerbated aortic calcification in niacin-treated CKD mice (Fig. 5C). Whole mount staining of aortas indicated SIRT1/SIRT6 inhibition abrogated niacin’s anti-calcific effects (Fig. 5D). Consistently, von Kossa (Fig. 5E) and Alizarin red S (Fig. 5F) staining of aortic section indicated that niacin-alleviated vascular calcification was blocked after SIRT1/SIRT6 inhibition. Furthermore, we observed significant upregulation of Runx2 and OPN of aortic artery after SIRT1 or SIRT6 inhibition (Fig. 5G). In all, these results demonstrated that the SIRT1 and SIRT6 signaling pathway are required in niacin-attenuated vascular calcification.

### Activation of autophagy is required for the inhibition of vascular calcification by niacin

**Fig. 6:**
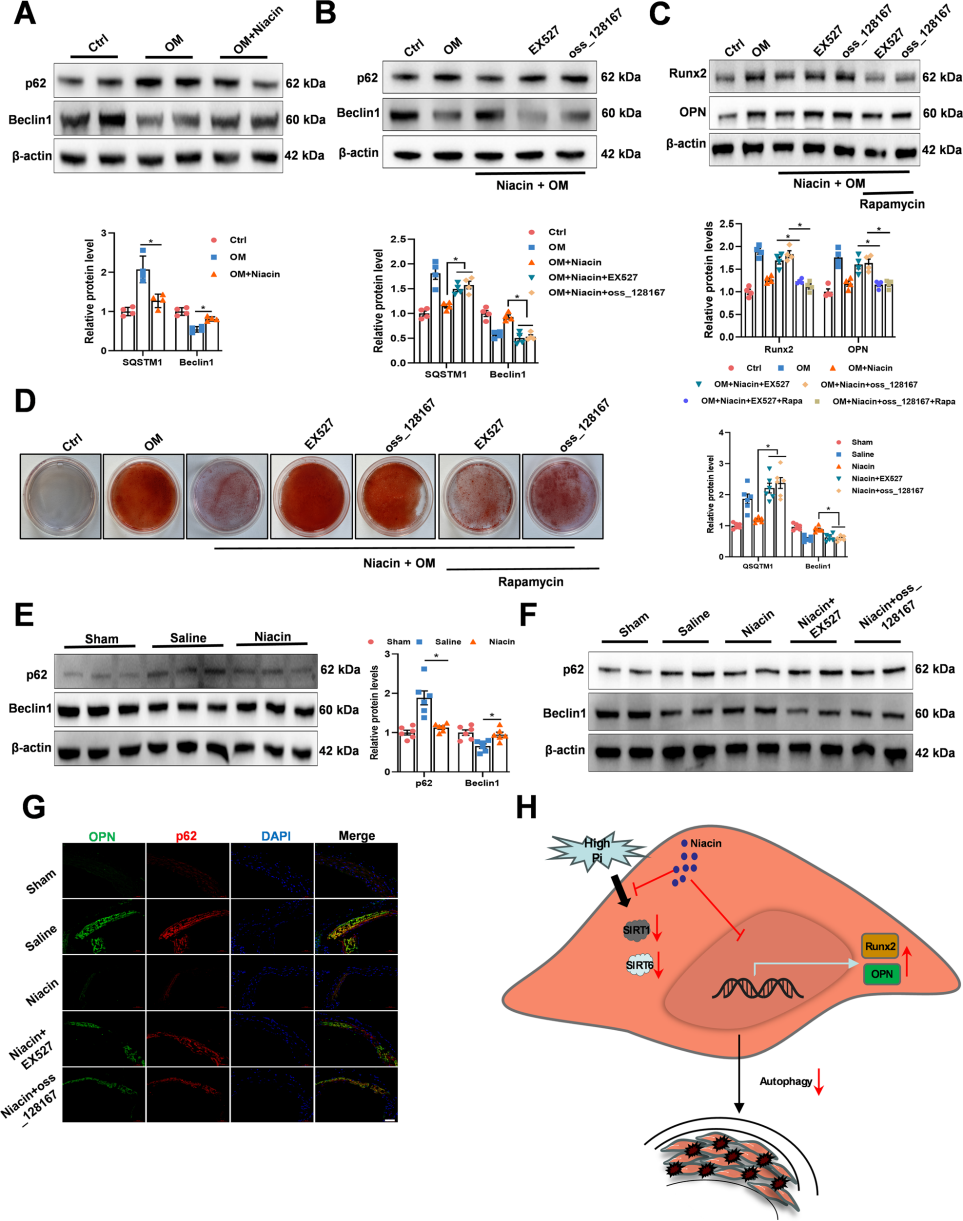
Niacin-mediated inhibition of vascular calcification through SIRT1 and SIRT6-mediated VSMC autophagy activation. **A** RASMCs were incubated with growth medium or calcifying medium with or without niacin. Autophagy-associated markers p62 and Beclin1 are examined by Western blot. **B** Representative western blots for the expression levels of p62 and Belcin1 after EX527 and OSS_128167 treatment. **C** Representative western blots for the expression levels of p62 and Belcin1 after EX527 and OSS_128167 treatment together with rapamycin (200 nM). **D** Representative images showing cells stained with Alizarin red solution at day 14. **E** and **F** Western blots for the expression level of vascular p62 and Beclin1. **G** Representative immunofluorescence images staining for p62 and OPN of aortic arteries. Scale bar = 50 μm. Data are shown as mean **±** SEM. * *P* < 0.05, ** *P* < 0.01. One-way ANOVA, Tukey’s HSD post hoc test. **H** Schematic diagram of the mechanism of niacin on vascular calcification. Under osteogenic conditions (e.g., hyperphosphatemia in CKD), SIRT1/SIRT6 expression is suppressed. Niacin restores SIRT1/SIRT6 levels and enhances autophagic flux, thereby inhibiting VSMC calcification.

Previous studies implicated autophagy in vascular calcification regulation, with both SIRT1 and SIRT6 signaling participating in this process [31–33]. We speculated that niacin may activate SIRT1/SIRT6-mediated autophagy in VSMC. Our results showed that OM treatment suppressed autophagy in VSMC, an effect partially reversed by niacin treatment (Fig. 6A). However, this impact was diminished after SIRT1 or SIRT6 inhibitor supplement, suggesting niacin activated VSMC autophagy through SIRT1/SIRT6 pathway (Fig. 6B). Rapamycin, a known autophagy enhancer, attenuated the pro-calcific effects of both EX527 and OSS_128167 in VSMC (Fig. 6C, D). In vivo, niacin restored autophagy impaired by CKD in vascular tissue (Fig. 6E). However, this effect was canceled after SIRT1 or SIRT6 inhibition (Fig. 6F, G). These results indicated that niacin treatment ameliorated vascular calcification via SIRT1 and SIRT6-mediated activation of VSMC autophagy (Fig. 6H).

## Discussion

Vascular calcification features ectopic mineral deposition of calcium and phosphate crystals in the tunica media and is considered as a complex and active process partially resembling bone mineralization [5, 6]. Vascular calcification increases major adverse cardiovascular events in CKD patients [34]. Despite recognition of multiple underlying mechanisms, limited effective therapy is available. In the present study, we demonstrated that niacin attenuated CKD and Vitamin D_3_-induced vascular calcification. To our knowledge, these data provide the first evidence that niacin treatment inhibits vascular calcification through SIRT1/SIRT6 pathway.

Shared mechanisms exist between aging and vascular calcification [35]. Intriguingly, previous studies have demonstrated that anti-aging agents such as resveratrol and irisin demonstrate protective effects against vascular calcification [36–38]. Niacin maintains endothelial nitric oxide, improves vascular aging [39], and has emerged as an antioxidant that exhibits cardiovascular protecting effect [40, 41]. RCS analysis further associates higher niacin intake with reduced abdominal aortic calcification, supporting its protective role. Moreover, we found that niacin treatment inhibited the calcification of RASMCs and significantly alleviates CKD and VD_3_ overload-induced vascular calcification in vivo. These data suggested that niacin may become a promising candidate for the prevention or treatment of vascular calcification in clinical applications since niacin is a dietary component with low toxicity and established efficacy.

SIRT1 and SIRT6, two members of the sirtuin family associated with cellular senescence, have been shown to inhibit vascular calcification. Their expression is reduced in calcified vessels [14, 42]. Activation of both SIRT1 and SIRT6 may become a promising clue for treatment of vascular calcification. Previously, niacin has been shown to activate SIRT1 in several studies [26, 39, 43]. Of note, we observed niacin treatment upregulated both SIRT1 and SIRT6 in calcifying VSMC. By using SIRT1 inhibitor or SIRT6 inhibitor, we found that either EX527 or OSS_128167 treatment canceled niacin diminished vascular calcification. Our data establish that both sirtuins mediate niacin’s protection against vascular calcification.

Accumulating evidence implicates autophagy as a critical regulator of VSMC calcification. Rapamycin, a classic autophagy activator, is reported to alleviate cell senescence, extend life span of animals, and block vascular calcification. SIRT1 and SIRT6 are reported to be regulators of autophagy [44, 45]. Notably, our results suggested that niacin significantly increased autophagy in VSMC, whereas EX527 or OSS_128167 treatment blocked it. However, rapamycin treatment markedly prevented EX527 or OSS_128167 induced vascular calcification. Collectively, our findings verified that niacin treatment inhibited vascular calcification via promoting SIRT1/SIRT6-mediated VSMC autophagy flux.

While our study verified that niacin treatment was effective in inhibiting vascular calcification, limitations still exist. Although we illustrated that the effects of niacin on SIRT1 and SIRT6 and autophagy under calcifying state, the precise regulatory mechanisms warrant further exploration. In addition, even we performed two different vascular calcification models to exclude the impact of niacin on CKD. The current animal models are still not good enough to simulate vascular calcification in clinical patients. Finally, additional experiment is required to confirm the role of niacin on arterial calcification in SIRT1 and SIRT6 conditional knockout mice.

In conclusion, we identified niacin as a novel inhibitor of vascular calcification. Furthermore, niacin decelerates vascular calcification via modulating SIRT1 and SIRT6 and enhancing VSMC autophagy. Dietary proper niacin uptake may serve as a hopeful strategy for the treatment of vascular calcification. Our study paves the way for clinical trials investigating niacin’s efficacy against vascular calcification in CKD patients.

## Material and Methods

A detailed description of the “Materials and Methods” is available in the Supplementary Material.

### Ethical statement

All methods were performed in accordance with the relevant guidelines and regulations. This study was conducted in accordance with the Declaration of Helsinki and was approved by the Ethics Committee of Nanjing First Hospital, Nanjing Medical University (Ethical approval number: No. KY20190530-05). Written informed consent was obtained from all participants prior to their inclusion in the study.

### Association between niacin and AAC based on NHANES analysis

National Health and Nutrition Examination Survey (NHANES) is a large-scale, nationwide cross-sectional survey aimed at assessing the nutritional and health status of the US population. The NHANES study uses a complex weighted sampling method to ensure the sample is representative. In this study, all analyses were conducted according to NHANES’s official data analysis guidelines. As AAC-related data is only available in the 2013–2014 survey cycle, we included only participants from the 2013–2014 cycle. Participants without niacin intake and AAC index data were excluded, resulting in a final sample size of 2897 participants.

Detailed baseline data for all participants were collected, including age, sex, race, education level, and annual household income, obtained directly from the statistical questionnaires. Experienced measurers conducted detailed measurements of each participant’s height and weight, and BMI was calculated to assess obesity status. Smoking and drinking statuses were self-reported by the participants. For hypertension diagnosis, experienced measurers took blood pressure readings for each participant, averaging the results of five measurements. An average systolic pressure greater than 140 mmHg and a diastolic pressure greater than 90 mmHg were considered indicative of hypertension. Additionally, participants reporting a history of hypertension or taking hypertension medications were also considered to have hypertension. All hematological data were obtained after participants had fasted for at least eight hours. Detailed measurements of participants’ lipid profiles and blood calcium and phosphorus levels were performed. To explore the relationship between niacin and abdominal aortic calcification (AAC), we used the RCS curve method, adjusting for confounding factors, to fit a curve illustrating the relationship between niacin intake and the risk of AAC.

### Animal studies

All animal experiments were approved by the Institutional Animal Care Committee at Nanjing First Hospital, Nanjing Medical University, and complied with the ARRIVE guidelines. Ten-week-old male C57BL/6J mice were purchased from GemPharmatech Co., Ltd (Nanjing, Jiangsu province, China). Mice were housed in a customized pathogen-free room with an ambient temperature of 25 °C and a humidity between 30% and 70% and were exposed to 12-h light–dark cycles and fed with rodent food and adequate water. All animals used were healthy and immune-normal. AD-induced (Xie Tong Sheng Wu, China) mice CKD model was established as previously described. VD_3_ overload-induced CV was performed by s.c injection of 100 µL VD3 (5.5 × 105 U/kg) (MCE, Shanghai, China) once a day in 16-week-old mice for three times as described. In some experiments, CKD mice were treated with EX527 (5 mg/kg/d, MCE, Shanghai, China) and OSS_128167 (10 mg/kg/d, MCE, Shanghai, China) by intraperitoneal injection for 4 weeks. EX527 was dissolved in 1% DMSO (in physiological saline) and then added to culture medium to reach a final concentration of 100 μM. OSS_128167 was dissolved in 1% DMSO (in physiological saline) and then added to PBS to reach a final concentration of 200 μM. Niacin was given via gavage at a dose of 600 mg/kg once a day at the beginning of adenine treatment.

### Statistical analysis

All results are presented as mean ± SEM and all statistical analysis was performed by using GraphPad Software (GraphPad Software, Inc., USA). Differences between two groups were analyzed by Student’s *t* test. Multiple group datasets were analyzed by one-way ANOVA followed by followed by Tukey post hoc tests. Biological experimental replicates between each group were shown in figure legends, and *P* < 0.05 was considered significant.

## Supplementary information

Supplemental material

Tables

Western Blot Gels

## Data Availability

The raw RNA-seq data in this study has been uploaded to the SRA database with accession number PRJNA1311765. All datasets analyzed in the study are available from the corresponding authors on reasonable request.
