# Supplementary material for: Niacin inhibits vascular calcification via modulating of SIRT1/SIRT6 signaling pathway
Source: Cell Death Discov. 2025 Dec 6;12:38. doi: 10.1038/s41420-025-02882-2 (PMC12827405; doi:10.1038/s41420-025-02882-2)
Supplement: Supplementary file 1 — Supplemental material [file 41420_2025_2882_MOESM1_ESM.docx]

**Supplementary Material**

**Material and Methods:**

**Cell culture**

Primary rat aortic smooth muscle cells were prepared using explanting method as previously described. Briefly, 7w mice were intraperitoneally euthanized using sodium pentobarbital (150 mg/kg) and aortic arteries were separated. Aortic segments were then cut into small pieces and grown on the culture dish with SMCM (Sciencell, #1101, Carlsbad, CA, USA) supplemented with 10% FBS (Sbjbio, China) and added into it at 37°C in a humidified incubator. 3 days later, primary vascular smooth muscle cells were migrated from the aortic explants. Cells at passage 4 to 8 were used for further study. In some experiments, cells were treated with niacin at different concentrations (10, 25, 50 mM) in the presence of calcifying medium. Cells were treated with EX527 (10 μM) or OSS_128167 (20 μM) in some experiments.

**Small interfering RNA (siRNA) infection**

Primary rat aortic smooth muscle cells were seeded in the 6-well plate. Cells were transfected with SIRT1 or SIRT6 siRNA (Gemagene, Shanghai, China) when it reached a density of 80%-90% by using Lipofectamine 3000Transfection Reagent (Thermo Fisher Scientific) according to the manufacturer’s instructions. Scramble siRNA was used as negative control. The efficient of siRNA was examined by immunoblotting.

**ALP activity assay**

Alkaline phosphate (ALP) activity was measured by using alkaline phosphate assay kit (Beyotime, #P0321S, Shanghai, China). Cells were collected in ice-cold 0.1% Triton X-100 PBS. Protein concentration was measured using a BCA Protein Kit (Yeasen, #20201ES76, Shanghai, China). Protein samples were mixed with p-nitrophenylphosphate (p-NPP) substrate then incubated in 37℃ for 10 min. The reaction was terminated with 3 M NaOH. ALP activity was measured at 405 nm and was calculated as unit/mg protein.

**Alizarin S red staining**

For cell staining, vascular smooth muscle cells were seeded in 6-well plate. After used medium and reagents were removed, cells were fixed using 4% paraformaldehyde (PFA) for 15 min at room temperature. After that, 1% alizarin red solution (Solabio, PH=4.2, #G1452, Beijing, China) were added for 10 min at room temperature. After disposing of the solutions, cells were rinsed with ddH_2_O to eliminate excess dye and images were taken under an inverted microscope, and the observers were blinded to the experimental groups.

For the whole mount of aorta staining, aortic arteries were separated then fixed in water-free ethanol for 24h. after that, arteries were stained with 0.003% alizarin red solution (Solabio, PH=8.3, #G1450, Beijing, China) in 1% potassium hydroxide overnight. The aortic arteries were then washed with ddH_2_O at room temperature for 10 min three times. Aortic arteries were then rinsed in 2% potassium hydroxide and photographed by using an inverted microscope, and the observers were blinded to the experimental groups.

**Calcium content assay**

Calcium content assay was performed using a calcium content assay kit (Leagene, #TC1023, Beijing, China) in accordance with the manufacturer’s instructions. Briefly, cell or tissues were homogenized and the supernatant was isolated via centrifugation. 200 microliter of Methyl thymol blue (MTB) solution was mixed with 2.5μl samples and then incubated at room temperature for 10 min. the absorbance was examined at 610 nm by using a microplate reader. BCA Protein Assay (Yeasen, #20201ES76, Shanghai, China) was performed to assess total protein concentration. The relative calcium content normalized to the protein concentration was marked as μg/mg protein.

**Prediction of niacin targeted proteins**

The SMILES formula of niacin was retrieved in the PubChem database (https://pubchem.ncbi.nlm.nih.gov/) and imported into the SwissTargetPrediction tool (http://swisstargetprediction.ch/) to predict the potential binding targets of niacin, and a total of 7 targets were obtained, and there was a possibility of binding.

**Molecular docking**

The molecule structure of niacin was obtained from the PubChem database, and the structure of SIRT family proteins were obtained from the AlphFold database (https://alphafold.ebi.ac.uk/). Then, the active pockets of these 7 proteins were predicted by the CavityPlus tool (http://pkumdl.cn:8000/cavityplus/index.php), and finally the niacin and these active pockets were docked by AutoDock Vina, and the one with the lowest binding energy was selected for visualization.

**RNA-sequencing analysis**

Aortic vessels were dissected from mice in the adenine diet (AD) and AD + niacin groups, respectively. RNA-sequencing and analysis were performed by Novogene, Inc. (Beijing, China). Differently expressed genes were generated by DEseq2 with fold change >2.0 and *P* < 0.05.

**Von-Kossa staining of aortic sections**

Aortic section von-Kossa staining was performed using a Von-Kossa Stain Kit (abcam, #ab150687, Cambridge, UK) according to the manufacturer’s protocols. In brief, paraffin aortic sections were deparaffinized and hydrate in distilled water. Then, section slides were incubated in Silver Nitrate Solution (5%) for 30 min with exposure to ultraviolet light. Distilled water was used to rinse the slides for 5 min. After that, sections were incubated with Sodium Thiosulfate Solution (5%) for 3 min. Next, aortic sections were rinsed in running water for 2 min followed by 2 changes of distilled water followed by incubation in Nuclear Fast Red Solution for 5 min. Then, aortic sections were rinsed in running water for 2 min followed by 2 changes of distilled water and dehydrate very quickly in 3 changes of fresh absolute alcohol and sealed with neutral resin (Sinopharm Chemical Reagent Co., Ltd, #10004160, Shanghai, China). The observers were blinded to the experimental groups.

**Western blotting analysis**

Cell or tissue were lysed with RIPA buffer containing proteinase (Yeasen, #20124ES03, Shanghai, China) and phosphatase inhibitors (Yeasen, #20109ES05, Shanghai, China). Then, protein concentration was determined using a BCA Protein Assay Kit (Yeasen, #20201ES76, Shanghai, China). 40 μg of protein was loaded in SDS-PAGE and then was transferred to PVDF membranes. Next, membranes were blocked in 5% defatted milk TBST solution followed by three times washing with TBST and incubation with primary antibodies diluted in Western Blot Primary Antibody Dilution Buffer (#WB500D, NCM Biotech, Suzhou, China) overnight at 4℃. The next day, membranes were washed with TBST followed by incubation with the accordant HRP-conjugated secondary antibody. The blots were visualized by the ECL image system and qualified using the image J software.

All the primary and secondary antibodies used in western blotting are listed as follows: anti-Runx2 (1:1000, #12556S, CST, MA), anti-Osteopontin (1:1000, #ab63856, Abcam, Cambridge, UK), anti-SIRT1 (1:1000, #13161-1-AP, Proteintech, Wuhan, China), anti-SIRT6 (1:1000, #67510-1-lg, Proteintech, Wuhan, China), anti-β-Actin (1:100000, #AC026, Abcolonal, Shanghai, China), anti-GAPDH (1:10000, #HA721136, HUABIO, Hangzhou, China), HRP-conjugated Goat anti-Rabbit IgG (H+L) (1:10000, #ZB-2301, ZSGB-BIO, Beijing, China), HRP-conjugated Goat anti-Mouse IgG (H+L) (1:10000, #ZB-2305, ZSGB-BIO, Beijing, China).

**Supplementary Figures**


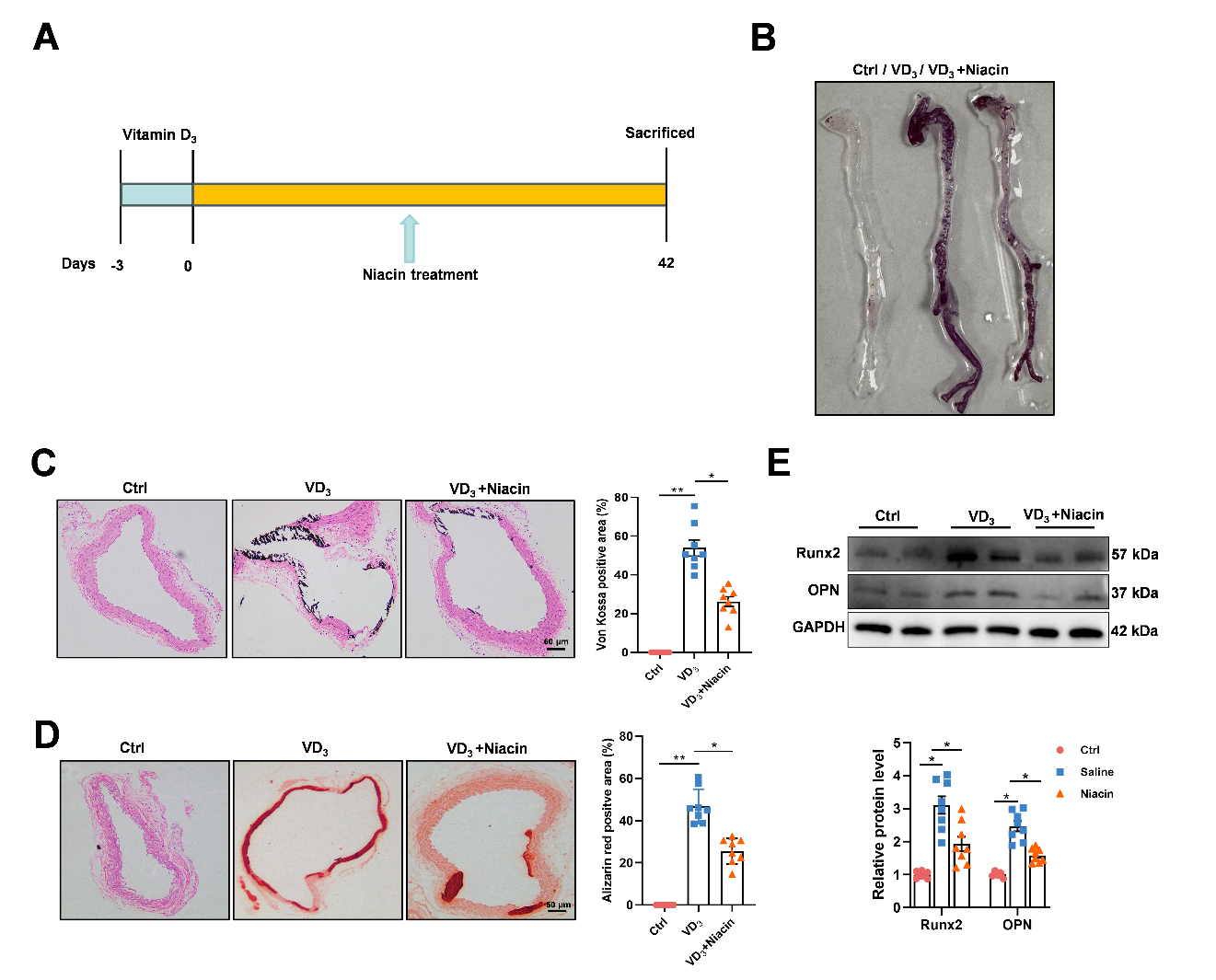


**Supplementary Figure S1.** **Niacin inhibits Vitamin D_3_ (VD_3_)-induced medial arterial calcification.** 12 week-year-old C57BL/6J mice were treated with Vitamin D_3_ (5.5×105 U/kg) for 3 consecutive days via subcutaneous injection, then subjected to niacin treatment for 6 weeks (n=8). (A) Scheme of the construction of the VD_3_-associated vascular calcification model and niacin supplement. (B) Mineral deposition was detected by alizarin red staining of whole mount of aorta. Representative images showing aortic arteries staining with alizarin red. (C and D) Representative images of alizarin red and von Kossa staining of aortic sections. Scale bar = 50 μm. (E) Representative western blot images for Runx2 and OPN expression in aortic vessels from different group. * *P* < 0.05, ** *P* < 0.01. One-way ANOVA followed by Tukey’s post hoc test.


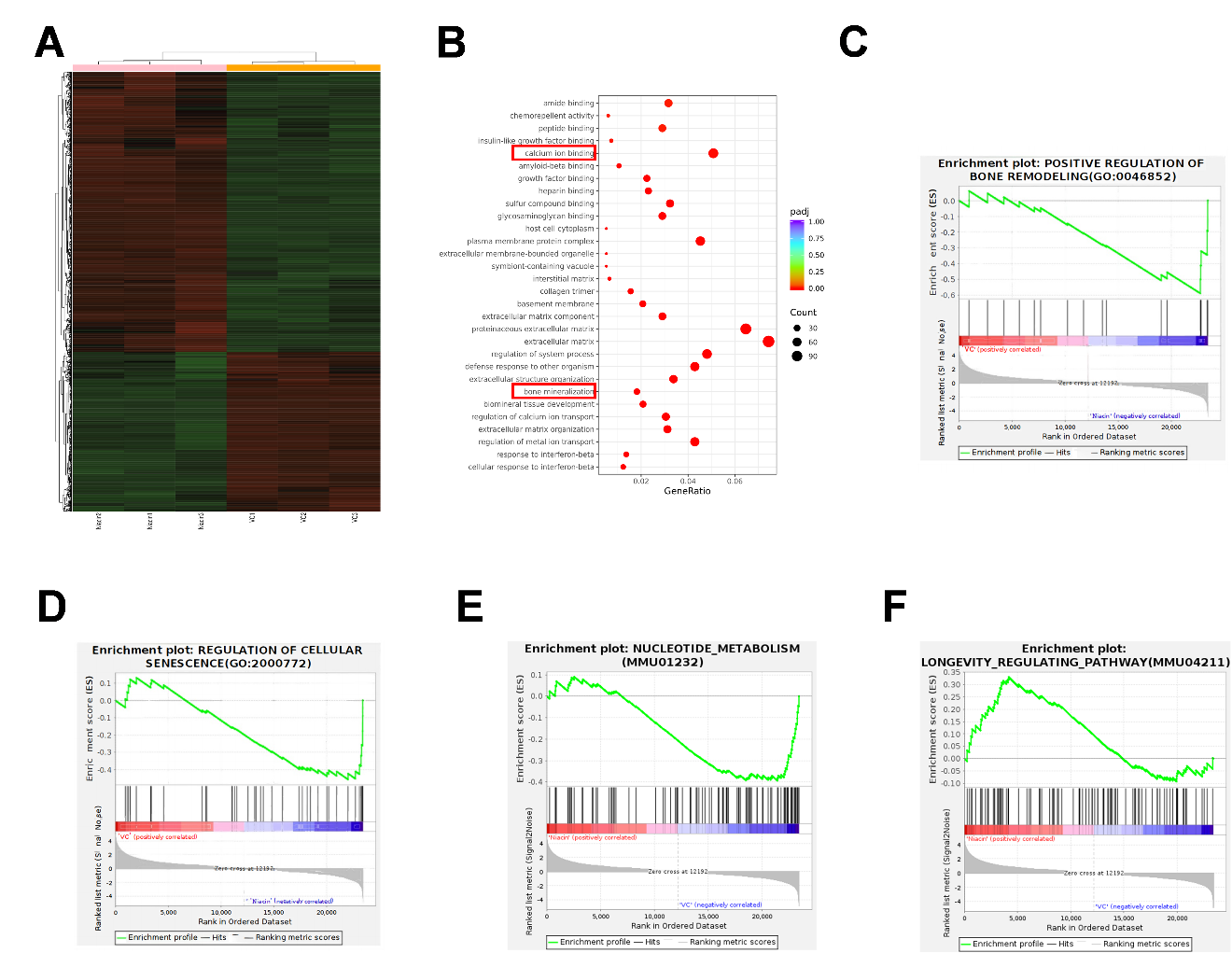


**Supplementary Figure S2. Analysis of whole transcriptome sequencing.** A. Heat map representing the differentially expressed genes (DEGs) from two groups. B: KEGG pathway enrichment analysis in downregulated genes in niacin-treated vessels compared with calcifying vessels. C-F: GSEA of regulated genes in niacin-treated vessels compared with calcifying vessels.


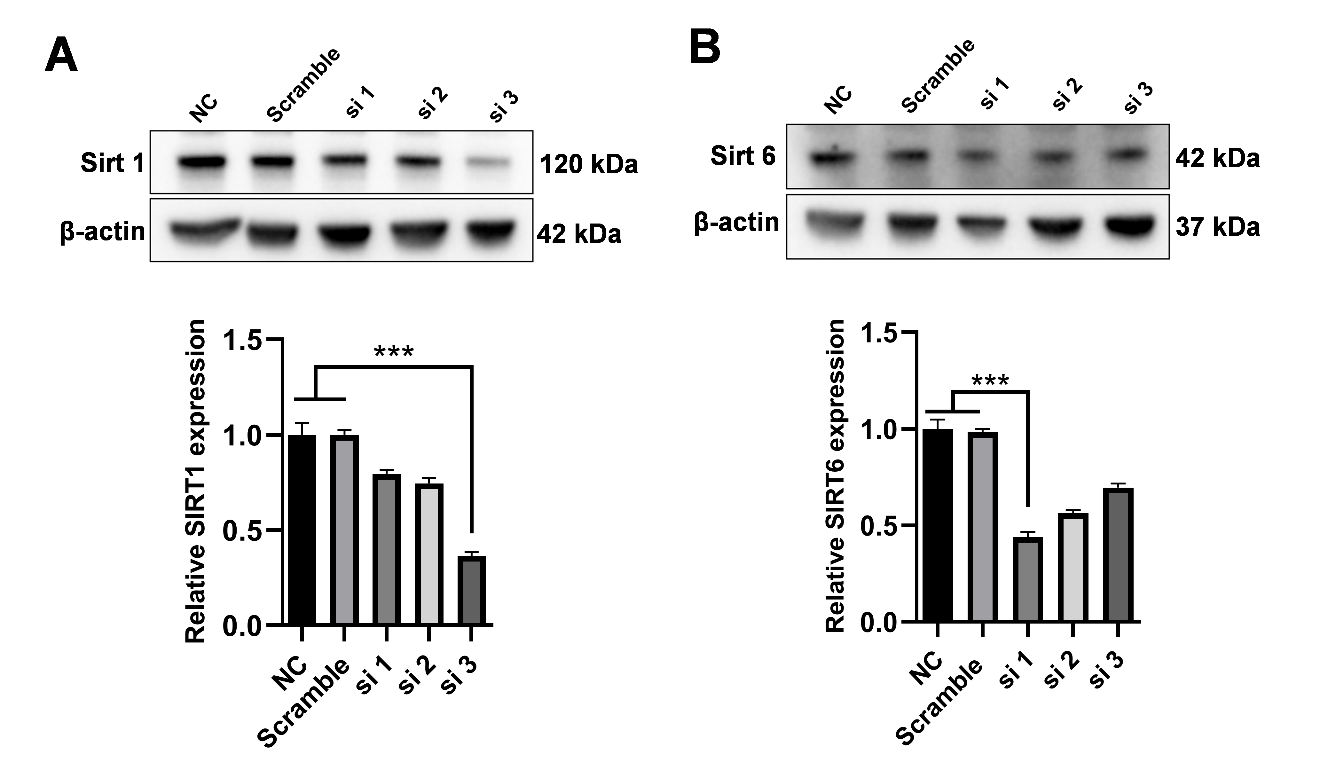


**Supplementary Figure S3. Examination of Sirt1 and Sirt6 siRNA.** Three different siRNAs targeting Sirt1 (A) or Sirt6 (B) were designed and transfected into MASMCs to verify the efficiency by immunoblotting. Data are shown as mean **±** SEM. * *P* < 0.05, ** *P* < 0.01, N.S. not significant. One-way ANOVA, Tukey's HSD post hoc test.
