# Supplementary material for: Niacin inhibits vascular calcification via modulating of SIRT1/SIRT6 signaling pathway
Source: Cell Death Discov. 2025 Dec 6;12:38. doi: 10.1038/s41420-025-02882-2 (PMC12827405; doi:10.1038/s41420-025-02882-2)
Supplement: Supplementary file 2 — Tables [file 41420_2025_2882_MOESM2_ESM.docx]

**Supplementary Tables**

**Supplementary Table S1. Baseline Characteristics of patients in this study.**

| **Variables** | **Overall**  **(n = 2897)** | **Non-AAC**  **(n = 2030)** | **AAC**  **(n = 867)** | ***P* value** |
| --- | --- | --- | --- | --- |
| **Age, years** |  |  |  | <0.001*** |
| **40-49 years** | 29.79 [25.24, 34.34] | 35.49 [33.62, 37.36] | 15.53 [10.36, 20.69] |  |
| **50-59 years** | 29.43 [25.32, 33.53] | 31.93 [28.57, 35.29] | 23.17 [18.24, 28.10] |  |
| **60-69 years** | 23.06 [18.88, 27.24] | 22.31 [19.73, 24.89] | 24.94 [20.47, 29.40] |  |
| **> 69 years** | 17.72 [15.74, 19.70] | 10.28 [8.41, 12.14] | 36.37 [30.88, 41.85] |  |
| **Sex-male, %** | 47.58 [41.27, 53.90] | 47.44 [45.03, 49.84] | 47.95 [44.71, 51.18] | 0.83 |
| **Race, %** |  |  |  | 0.34 |
| **White** | 88.65 [76.64, 100.67] | 88.29 [85.97, 90.61] | 89.57 [87.01, 92.13] |  |
| **Other** | 11.35 [9.70, 12.99] | 11.71 [9.39, 14.03] | 10.43 [7.87, 12.99] |  |
| **Education level, %** |  |  |  | 0.74 |
| **High school or above** | 95.30 [83.27, 107.33] | 95.42 [93.93, 96.91] | 95.01 [92.71, 97.31] |  |
| **Below high school** | 4.70 [3.46, 5.93] | 4.58 [3.09, 6.07] | 4.99 [2.69, 7.29] |  |
| **Income, %** |  |  |  | <0.001*** |
| **≥ 2000$** | 13.78 [9.77, 17.79] | 12.77 [9.22, 16.32] | 17.86 [12.49, 23.23] |  |
| **< 2000$** | 83.08 [71.55, 94.61] | 87.23 [83.68, 90.78] | 82.14 [76.77, 87.51] |  |
| **BMI** |  |  |  | 0.01* |
| **Normal weight** | 26.43 [22.44, 30.42] | 26.25 [24.85, 27.65] | 27.21 [23.58, 30.85] |  |
| **Over weight** | 36.09 [31.60, 40.58] | 38.26 [34.96, 41.56] | 31.10 [27.64, 34.55] |  |
| **Obesity** | 37.13 [31.62, 42.63] | 35.49 [32.35, 38.64] | 41.69 [37.95, 45.43] |  |
| **Smoking, %** |  |  |  | 0.001** |
| **Current** | 17.04 [14.06, 20.02] | 15.66 [13.69, 17.64] | 20.52 [14.73, 26.31] |  |
| **Former** | 28.31 [23.37, 33.25] | 25.58 [22.70, 28.46] | 35.18 [31.65, 38.71] |  |
| **Never** | 54.61 [47.62, 61.61] | 58.75 [56.39, 61.12] | 44.30 [36.99, 51.62] |  |
| **Alcohol user, %** |  |  |  | 0.16 |
| **Heavy** | 13.77 [11.86, 15.68] | 13.68 [11.69, 15.67] | 15.34 [11.40, 19.28] |  |
| **Low to moderate** | 55.58 [46.80, 64.36] | 58.98 [52.99, 64.96] | 52.57 [48.94, 56.19] |  |
| **Never** | 27.92 [22.49, 33.36] | 27.34 [21.43, 33.25] | 32.10 [28.99, 35.20] |  |
| **Hypertension, %** | 50.52 [45.38, 55.67] | 44.69 [41.67, 47.71] | 65.13 [59.43, 70.84] | <0.001*** |
| **eGFR, mL/min/1.73m^2^** | 84.18 [83.22, 85.13] | 86.62 [85.56, 87.68] | 78.04 [75.76, 80.32] | <0.001*** |
| **Albumin, g/dL** | 4.25 [4.23, 4.27] | 4.26 [4.23, 4.28] | 4.24 [4.21, 4.27] | 0.44 |
| **Albuminuria, mg/L** | 33.47 [25.09, 41.85] | 30.48 [19.46, 41.51] | 41.01 [32.59, 49.43] | 0.15 |
| **Serum calcium, mg/dL** | 9.45 [9.43, 9.48] | 9.44 [9.41, 9.47] | 9.48 [9.43, 9.54] | 0.18 |
| **Serum phosphorus, mg/dL** | 3.80 [3.77, 3.83] | 3.80 [3.76, 3.84] | 3.81 [3.76, 3.85] | 0.79 |
| **HbA1c, %** | 5.76 [5.72, 5.81] | 5.71 [5.65, 5.76] | 5.91 [5.83, 5.98] | <0.001*** |
| **FBG, mg/Dl** | 107.65 [105.70, 109.60] | 106.40 [104.25, 108.56] | 110.62 [106.53, 114.70] | 0.09 |
| **FBI, pmol/L** | 72.36 [64.03, 80.69] | 73.46 [63.26, 83.66] | 69.72 [59.39, 80.06] | 0.59 |
| **TC, mmol/L** | 5.06 [5.04, 5.09] | 5.08 [5.02, 5.15] | 5.01 [4.93, 5.10] | 0.33 |
| **TG, mmol/L** | 1.81 [1.74, 1.88] | 1.77 [1.69, 1.86] | 1.89 [1.81, 1.98] | 0.04* |
| **HDL-C, mmol/L** | 1.42 [1.40, 1.43] | 1.43 [1.42, 1.45] | 1.37 [1.33, 1.41] | 0.004** |
| **LDL-C, mmol/L** | 2.99 [2.94, 3.04] | 2.99 [2.92, 3.06] | 2.99 [2.94, 3.04] | 0.98 |

Continuous variables are presented as the mean [95% CI], category variables are presented as the proportion [95% CI].

Abrevation: AAC, abdominal aortic calcification; BMI, body mass index; CI, confidence interval; eGFR, estimated glomerular filtration rate; ePWV, estimated pulse wave velocity; FBG, fasting blood glucose; FBI, fasting blood insulin; HbA1c, glycated hemoglobin; HDL-C, high-density lipoprotein cholesterol; TC, total cholesterol; TG, triglycerides; LDL-C, low-density lipoprotein cholesterol; BRI, body round index.

***** *P* value<0.05, ****** *P* value<0.01, *** *P* value<0.001.

**Supplementary Table S2. Basic information of mice.**

| **Group** | | **Body**  **Weight(g)** | **Serum Cr**  **(μmol/L)** | **Serum P**  **(mmol/L)** | **Serum Ca**  **(mmol/L)** | **BUN**  **(mmol/L)** |
| --- | --- | --- | --- | --- | --- | --- |
| **Shame**  **(n=8)** | **0 week** | **26.23±0.62** | **53.00±1.93** | **2.18±0.63** | **2.14±0.08** | **7.97±0.27** |
|  | **4 weeks** | **25.65±0.83** | **57.64±2.45** | **2.22±0.61** | **2.21±0.13** | **8.57±0.21** |
|  | **8 weeks** | **27.38±0.62** | **60.84±1.89** | **2.38±0.67** | **2.32±0.22** | **9.78±0.31** |
|  | **12 weeks** | **27.68±0.55** | **70.70±1.20** | **2.45±0.62** | **2.42±0.15** | **10.83±0.19** |
| **CKD**  **(n=8)** | **0 week** | **25.34±0.88** | **53.28±2.57** | **2.15±0.56** | **2.09±0.12** | **8.34±0.23** |
|  | **4 weeks** | **22.86±0.57*** | **174.60±4.47**** | **3.36±0.83*** | **2.19±0.22** | **25.15±0.94*** |
|  | **8 weeks** | **19.04±0.32*** | **216.20±6.13**** | **3.90±0.52*** | **2.34±0.52** | **35.33±1.77**** |
|  | **12 weeks** | **16.51±0.46*** | **278.50±4.09**** | **4.45±0.12*** | **2.51±0.64*** | **51.18±0.84**** |
| **CKD+Niacin**  **(n=8)** | **0 week** | **25.08±0.66** | **53.50±2.31** | **2.18±0.71** | **2.12±0.10*** | **8.29±0.21** |
|  | **4 weeks** | **22.76±0.60*** | **168.50±6.26**** | **3.36±0.75*** | **2.29±0.42** | **21.43±0.76*** |
|  | **8 weeks** | **22.56±0.53*#** | **218.60±6.23**** | **3.54±0.58*** | **2.28±0.37*** | **31.59±1.57**#** |
|  | **12 weeks** | **22.00±0.69*#** | **237.70±6.51**#** | **3.68±0.11*#** | **2.34±0.38** | **39.67±0.84**#** |

Data are shown as mean**±**SEM. BUN: blood urea nitrogen; Cr: creatinine; P: phosphate; Ca: calcium. ***** *P* value<0.05, ****** *P* value<0.01, vs. the age-marched controls. # *P*<0.05, ## *P*<0.01, vs. the aged-marched CKD group.

**Supplementary Table S3. Basic information of mice.**

| **Group** | | **Body**  **Weight(g)** | **Serum Cr**  **(μmol/L)** | **Serum P**  **(mmol/L)** | **Serum Ca**  **(mmol/L)** | **BUN**  **(mmol/L)** |
| --- | --- | --- | --- | --- | --- | --- |
| **Shame**  **(n=8)** | **0 week** | **25.78±0.57** | **52.88±1.28** | **2.12±0.54** | **2.12±0.02** | **7.74±0.31** |
|  | **4 weeks** | **26.44±0.72** | **60.21±1.57** | **2.19±0.48** | **2.24±0.12** | **8.64±0.57** |
|  | **8 weeks** | **27.23±0.52** | **64.57±2.47** | **2.36±0.19** | **2.31±0.07** | **10.41±0.17** |
|  | **12 weeks** | **28.11±0.43** | **73.00±2.24** | **2.38±0.49** | **2.41±0.11** | **11.08±0.53** |
| **CKD**  **(n=8)** | **0 week** | **24.78±0.72** | **53.74±1.45** | **2.52±0.28** | **2.09±0.13** | **8.10±0.52** |
|  | **4 weeks** | **21.73±0.58*** | **168.47±3.73**** | **3.34±0.59*** | **2.21±0.07** | **27.14±1.01*** |
|  | **8 weeks** | **20.01±0.72*** | **215.78±2.47**** | **3.78±0.83*** | **2.37±0.14*** | **33.68±2.17**** |
|  | **12 weeks** | **18.53±0.63*** | **280.48±4.24**** | **4.58±0.27*** | **2.48±0.12*** | **53.17±1.55**** |
| **CKD+Niacin**  **(n=8)** | **0 week** | **25.33±0.54*#** | **54.25±2.11** | **2.20±0.52** | **2.08±0.09** | **8.05±0.78** |
|  | **4 weeks** | **21.23±0.44*#** | **157.56±4.58**** | **3.28±0.77*** | **2.25±0.13** | **19.57±1.27*** |
|  | **8 weeks** | **22.14±0.78*** | **208.50±6.54**** | **3.47±0.82*** | **2.27±0.20*** | **30.28±0.56*** |
|  | **12 weeks** | **21.23±0.35*#** | **225.54±5.87**#** | **3.58±0.28*#** | **2.32±0.04*#** | **37.54±0.59*#** |
| **CKD+Niacin+**  **EX527**  **(n=7)** | **0 week** | **26.47±0.85** | **51.79±2.15** | **2.15±0.28** | **2.08±0.06** | **7.55±0.51** |
|  | **4 weeks** | **22.27±0.14*** | **166.57±3.35**** | **3.52±0.29*** | **2.23±0.11** | **26.87±1.31*** |
|  | **8 weeks** | **20.73±0.15*** | **224.45±4.25**** | **3.82±0.32*** | **2.32±0.04** | **35.74±2.03*#** |
|  | **12 weeks** | **17.23±0.74*** | **279.51±7.56**** | **4.28±1.20*** | **2.44±0.12** | **46.57±0.38**#** |
| **CKD+Niacin+**  **oss_128167**  **(n=7)** | **0 week** | **24.73±0.72** | **55.54±2.14** | **2.18±0.71** | **2.10±0.09** | **7.68±0.28** |
|  | **4 weeks** | **21.27±0.47*** | **178.58±3.24**** | **3.68±0.87*** | **2.21±0.12** | **27.44±1.08*** |
|  | **8 weeks** | **19.88±0.67*** | **219.57±3.47**** | **3.82±0.57*** | **2.42±0.12#** | **37.74±1.07**#** |
|  | **12 weeks** | **18.22±0.58*** | **288.87±8.57**** | **4.24±1.05*** | **2.47±0.22*#** | **54.87±1.05*#** |

Data are shown as mean**±**SEM. BUN: blood urea nitrogen; Cr: creatinine; P: phosphate; Ca: calcium. ***** *P* value<0.05, ****** *P* value<0.01, vs. the age-marched controls. # *P*<0.05, ## *P*<0.01, vs. the aged-marched CKD group.

**Supplementary Table S4. Basic information of mice.**

| **Group** | | **Body**  **Weight(g)** | **Serum Cr**  **(μmol/L)** | **Serum P**  **(mmol/L)** | **Serum Ca**  **(mmol/L)** | **BUN**  **(mmol/L)** |
| --- | --- | --- | --- | --- | --- | --- |
| **Shame**  **(n=8)** | **0 week** | **27.03±0.32** | **61.38±3.21** | **2.25±0.21** | **2.23±0.12** | **8.81±0.24** |
|  | **6 weeks** | **31.55±0.46** | **68.86±2.57** | **2.34±0.41** | **2.32±0.09** | **10.15±0.64** |
| **VD_3_**  **(n=7)** | **0 week** | **26.98±0.47** | **60.94±2.87** | **2.18±0.41** | **2.20±0.07** | **9.04±0.35** |
|  | **6 weeks** | **29.03±0.23*** | **80.37±1.89*** | **3.36±0.33*** | **2.35±0.12*** | **18.85±0.87*** |
| **VD_3_+Niacin**  **(n=8)** | **0 week** | **25.78±0.57** | **62.14±2.56** | **2.23±0.34** | **2.24±0.11** | **8.97±0.47** |
|  | **6 weeks** | **30.07±0.81*** | **82.14±2.01*** | **3.45±0.21*** | **2.33±0.07*** | **20.38±1.03*** |

Data are shown as mean**±**SEM. BUN: blood urea nitrogen; Cr: creatinine; P: phosphate; Ca: calcium. ***** *P* value<0.05, ****** *P* value<0.01, vs. the age-marched controls. # *P*<0.05, ## *P*<0.01, vs. the aged-marched CKD group.
