# Supplementary material for: Niacin inhibits vascular calcification via modulating of SIRT1/SIRT6 signaling pathway
Source: Cell Death Discov. 2025 Dec 6;12:38. doi: 10.1038/s41420-025-02882-2 (PMC12827405; doi:10.1038/s41420-025-02882-2)
Supplement: Supplementary file 3 — Western Blot Gels [file 41420_2025_2882_MOESM3_ESM.docx]

Figure2A

Ctrl/OM/0.1mM/0.2mM/0.5mM (RASMCs)

**Runx2**




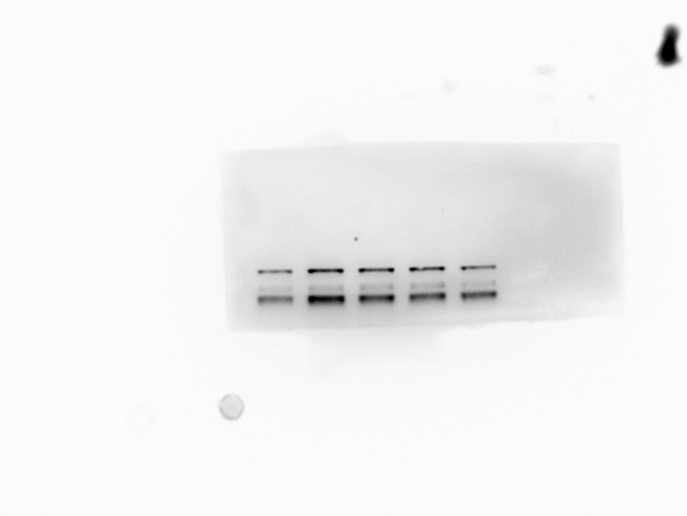


**OPN**




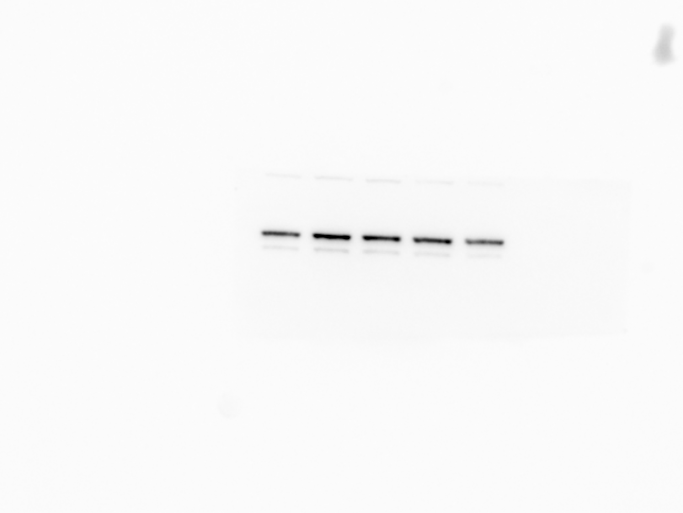


**β-actin**




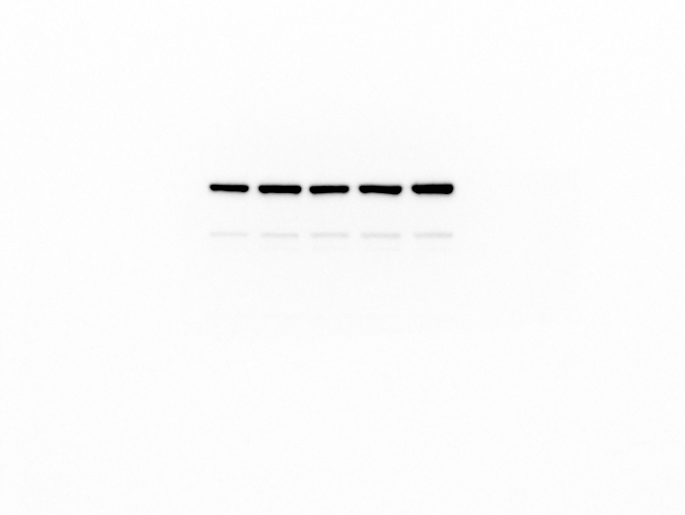


**Gels cut before and after**


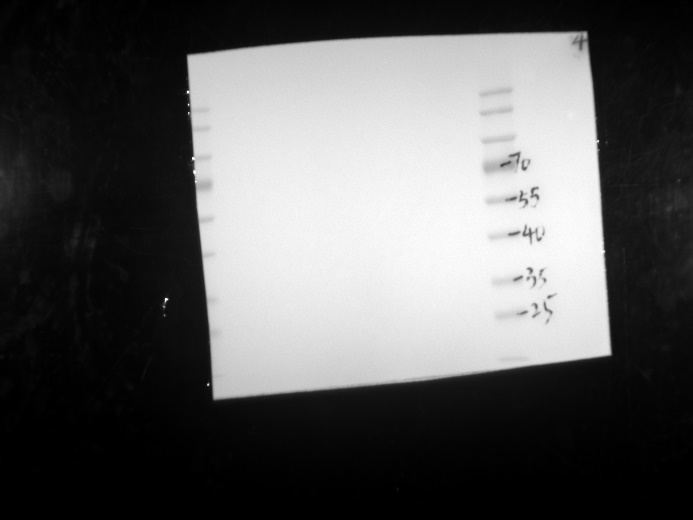

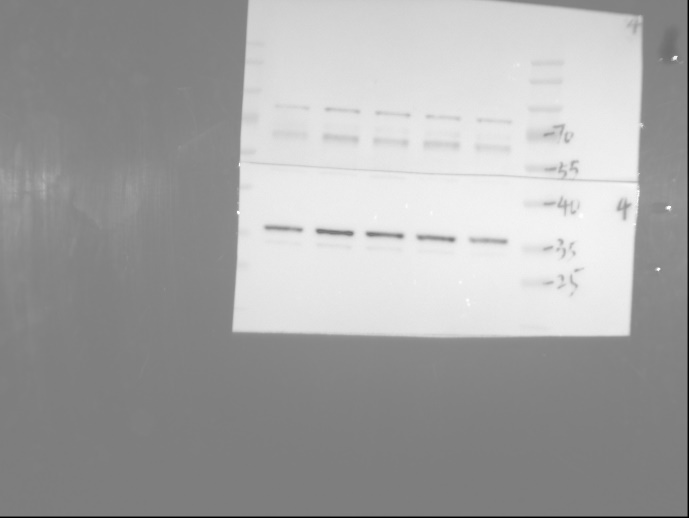


Figure 3F

Ctrl/OM/OM+Niacin (RASMCs)

**Runx2**




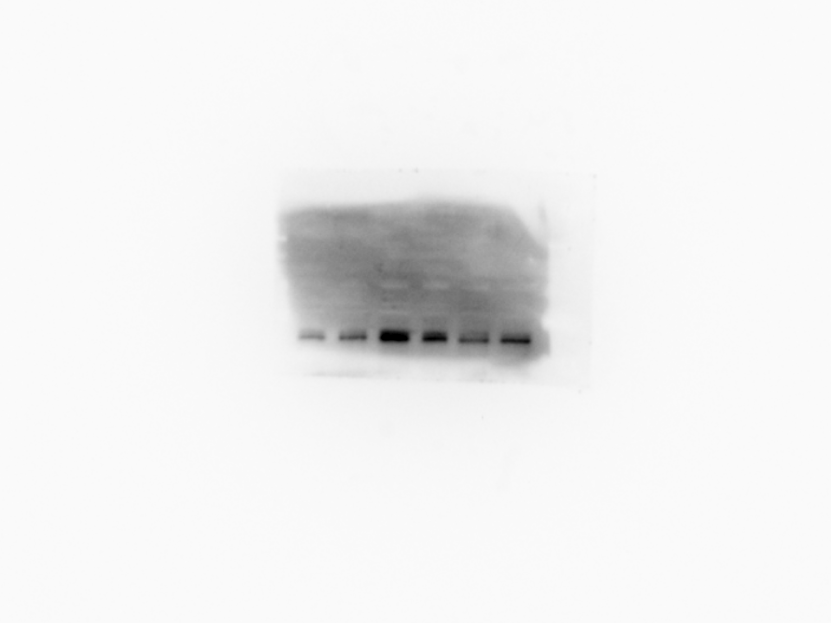


**OPN**




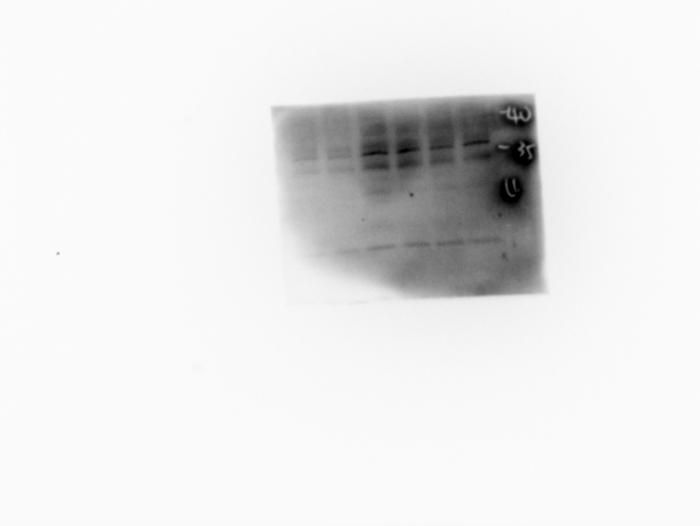


**β-actin**




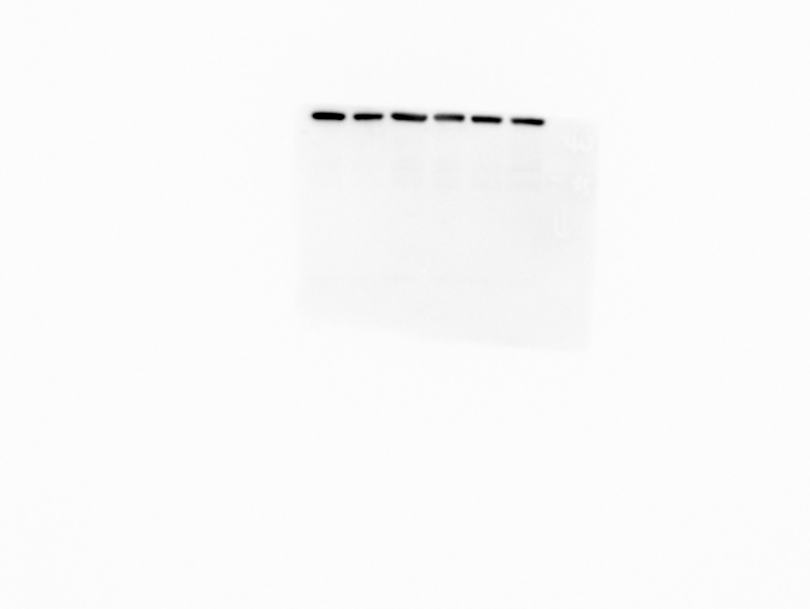



**Gels cut before and after**


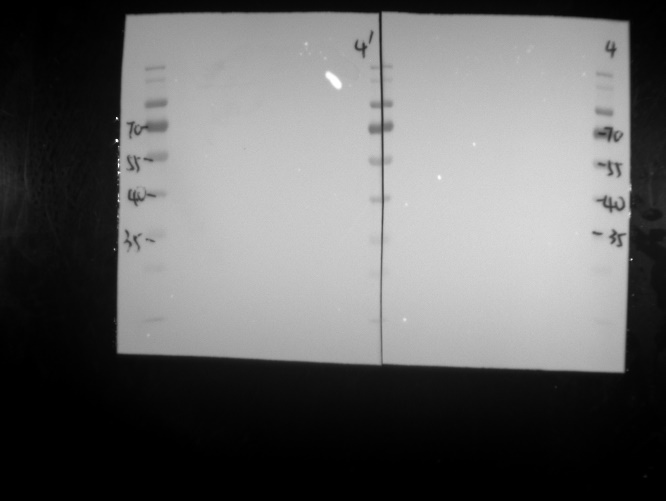


Figure 4D

Ctrl/OM/OM+Niacin (RASMCs)

**Sirt1**




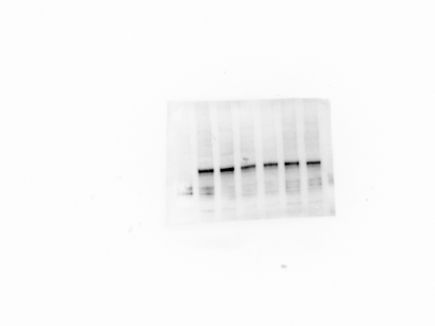


**Sirt6**




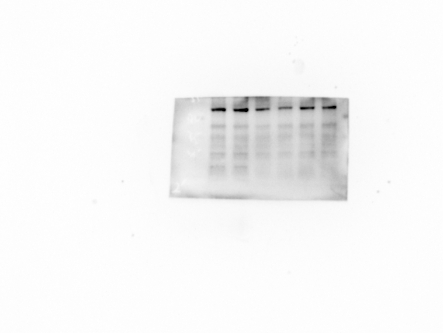


**GAPDH**




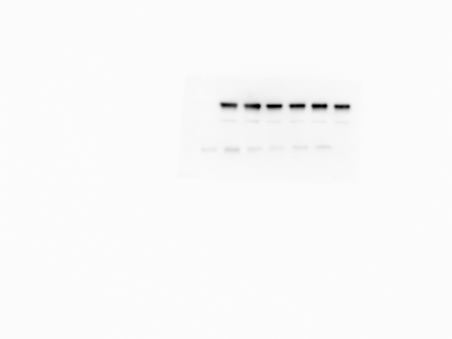


**Gels cut before and after**


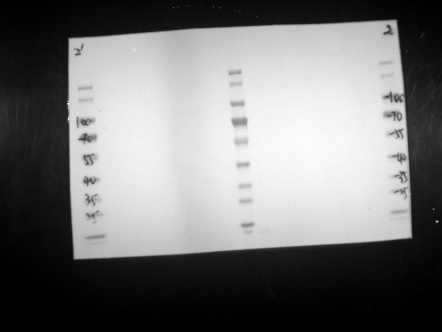




Figure4E

Ctrl/OM/OM+Niacin/OM+Niacin+EX527/OM+Niacin+oss_128167 (RAMSCs)

**Runx2**




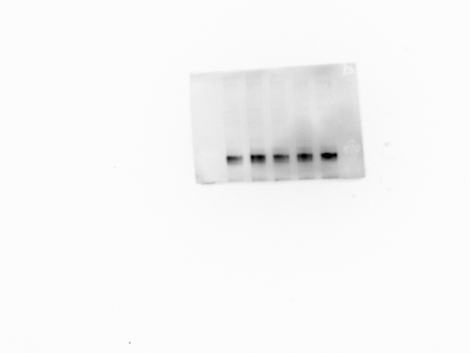


**OPN**




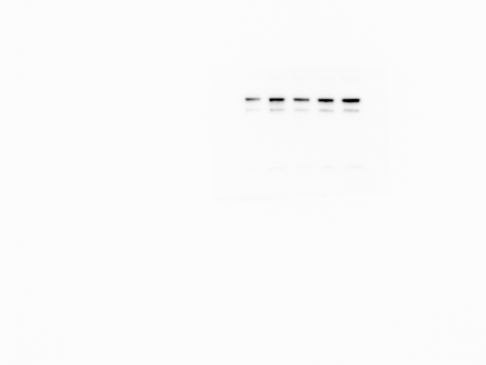


**β-actin**


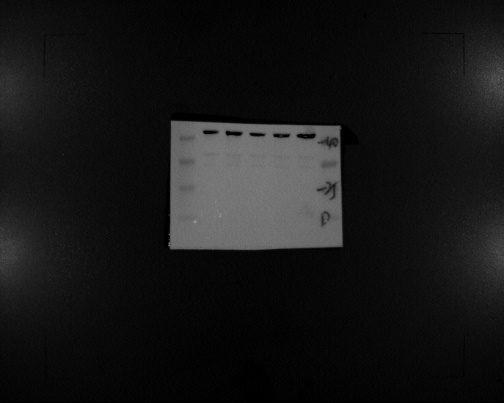

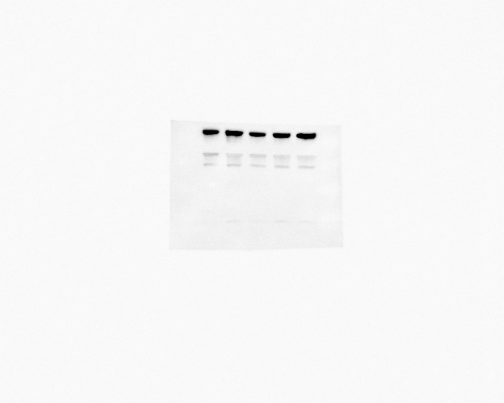


**Gels**


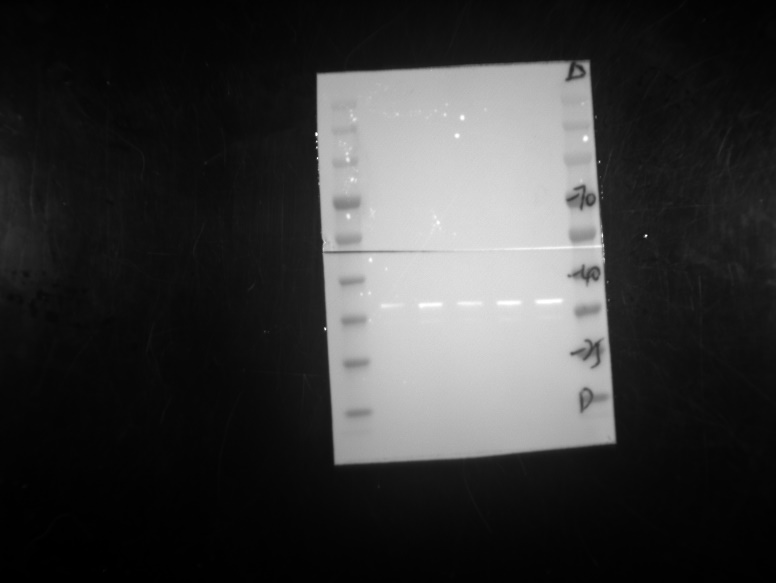


Figure4 I

Ctrl/OM/OM+Niacin/OM+Niacin+siSirt1/OM+Niacin+siSirt6 (RASMCs)

**Runx2**




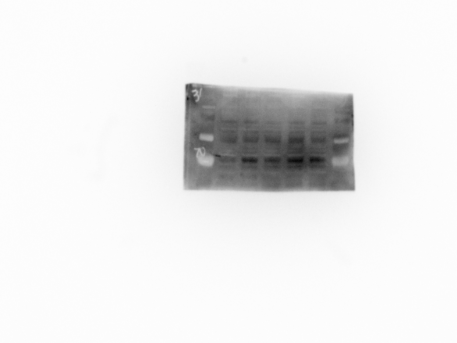


**OPN**




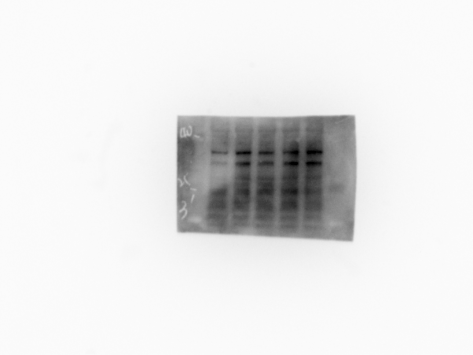


**Sirt1**




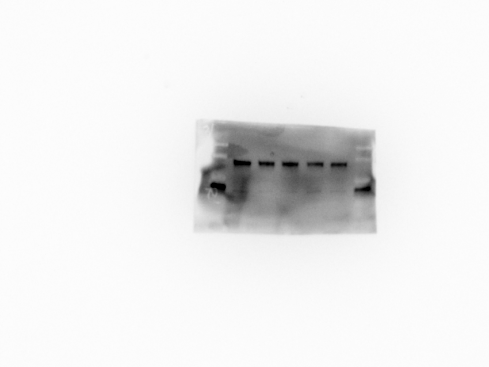


**Sirt6**




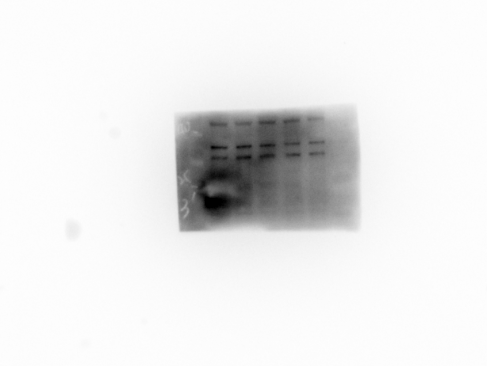


**GAPDH**




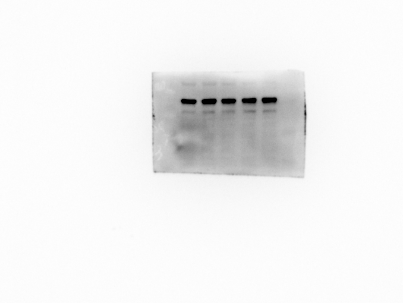


**Gels cut before and after**


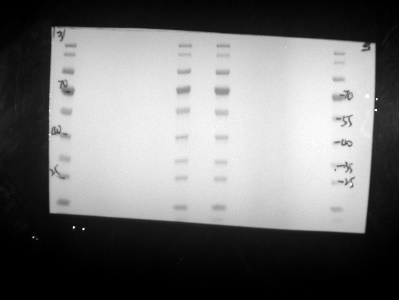

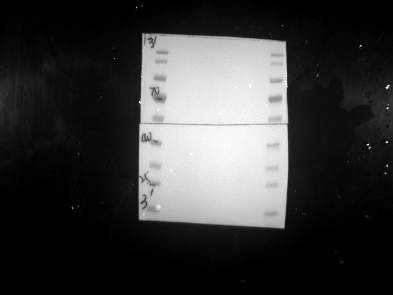


Figure5B

Shame/Saline/Niacin Vascular

**Sirt1**

**


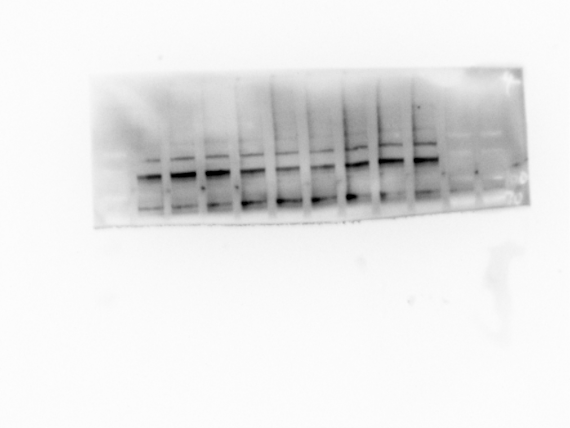
**

**Sirt6**

**


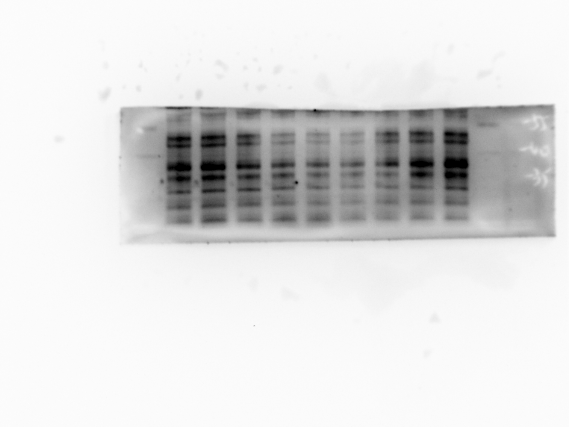
**

**GAPDH**




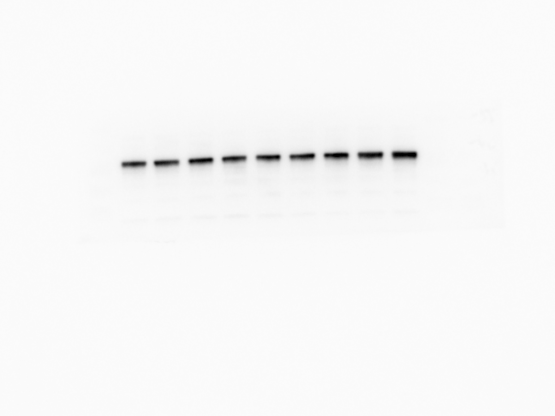


**Gels cut before and after**

**
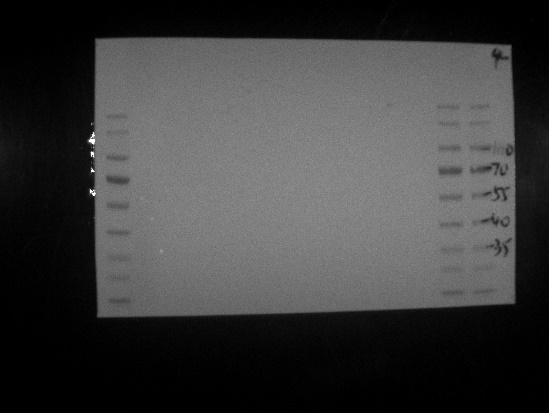

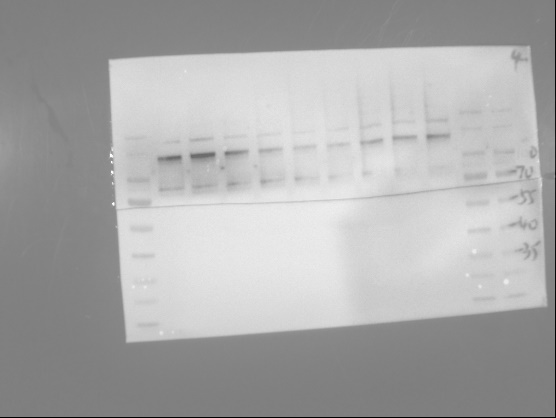
**

Figure5G

Sham/Saline/Niacin/Niacin+EX527/Niacin+ oss_128167 Vascular

**Runx2**




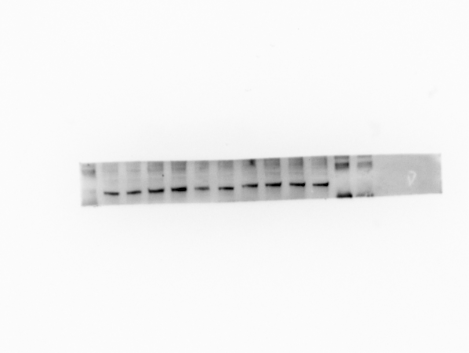


**OPN**


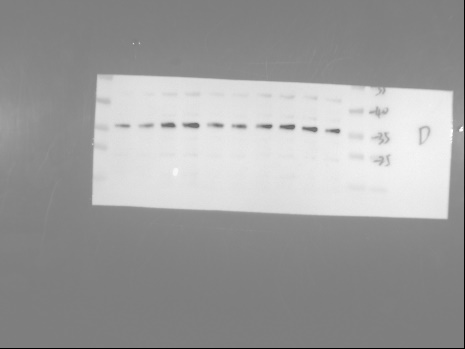

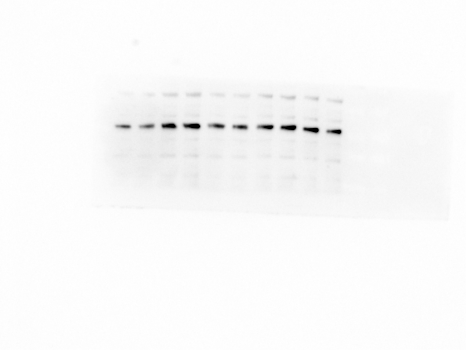


**β-actin**




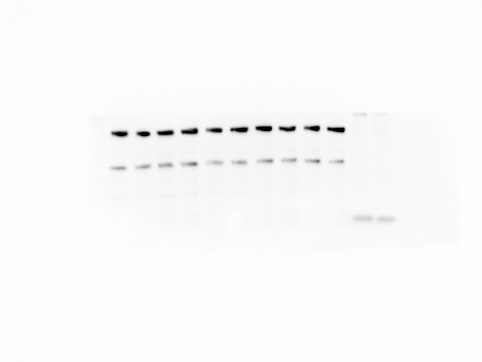


**Gels cut before and after**


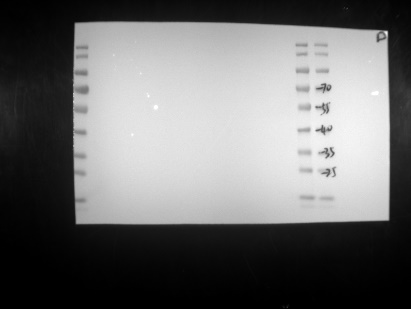

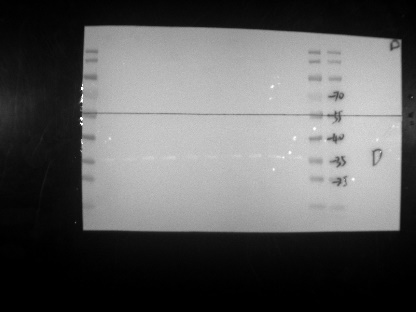


Figure6A

Ctrl/OM/OM+Niacin (RASMCs)

**QSQTM1**




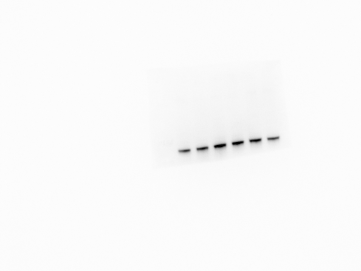


**β-actin**


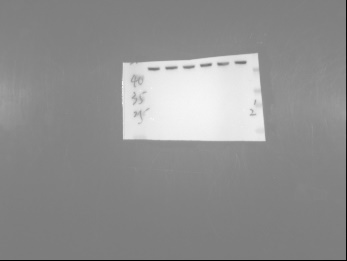

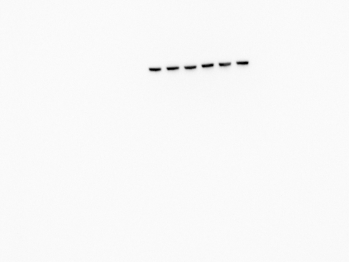


**Gels cut before and after**


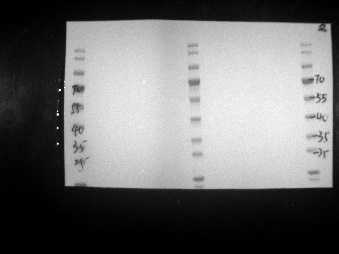

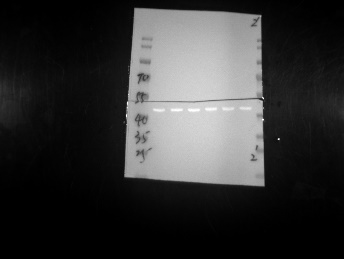


**Beclin1**


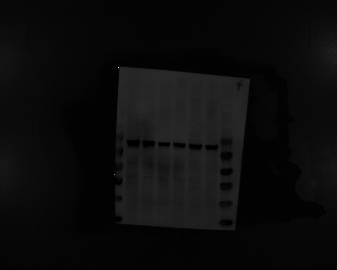

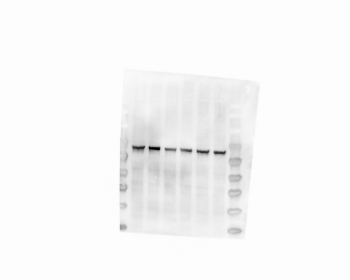


**GAPDH**


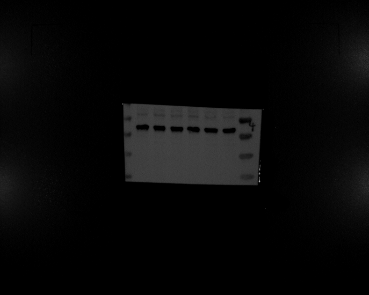

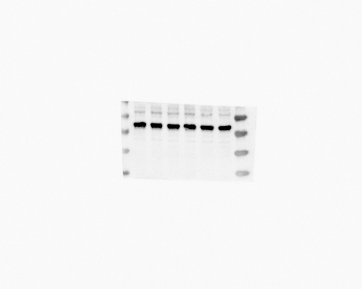


**Gels cut before and after**


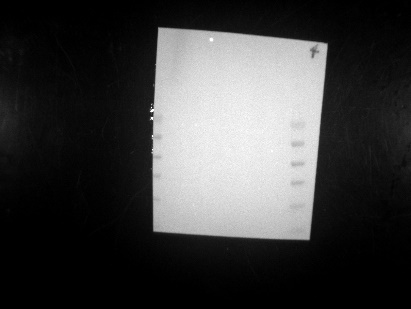

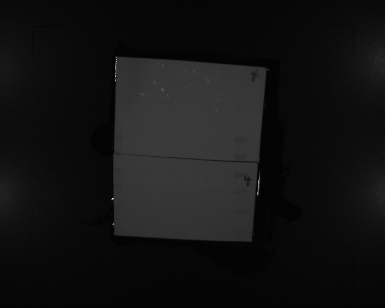


Figure6B

Ctrl/OM/OM+Niacin/OM+Niacin+EX527/OM+Niacin+oss_128167 (RAMSCs)

**SQSTM1**


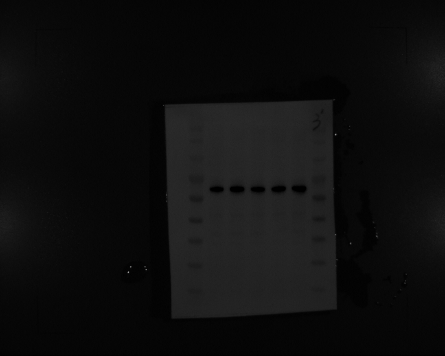

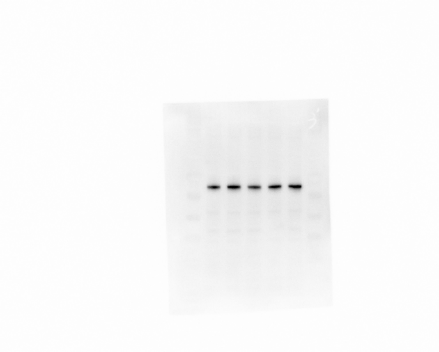


**β-actin**


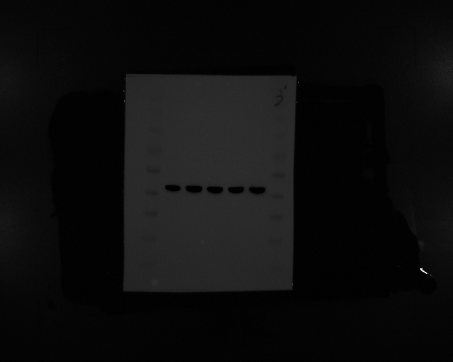

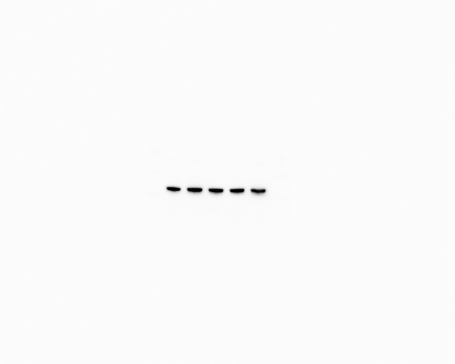


**Beclin1**




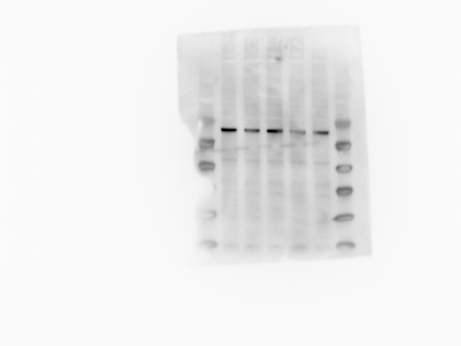


**GAPDH**


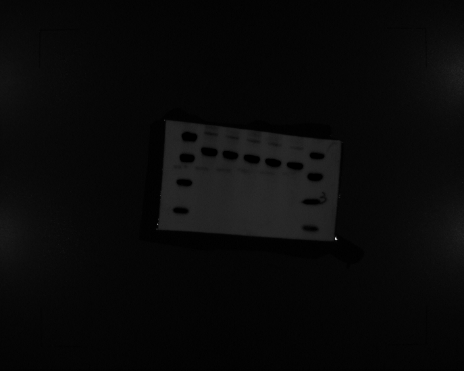

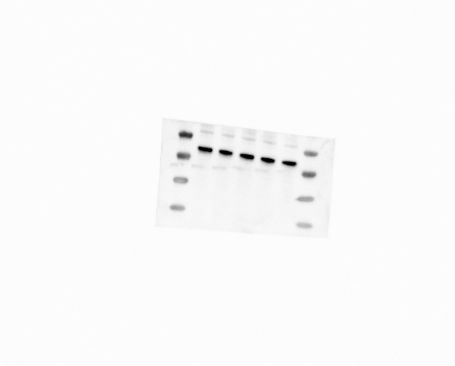


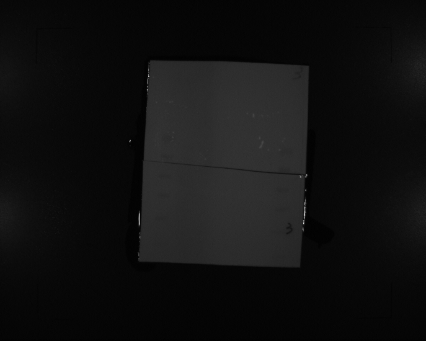


Figure6C

Ctrl/OM/OM+Niacin/OM+Niacin+EX527/OM+Niacin+oss_128167/ OM+Niacin+EX527+Rapamycin/OM+Niacin+oss_128167+Rapamycin

**Runx2**


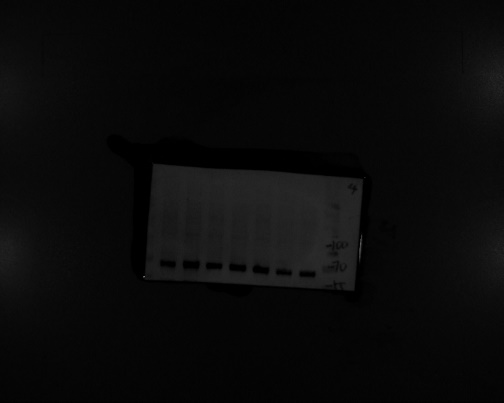

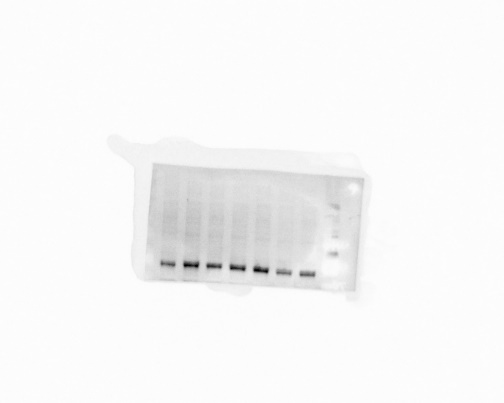


**OPN**


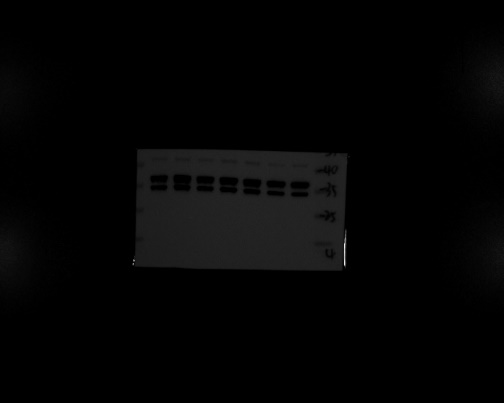

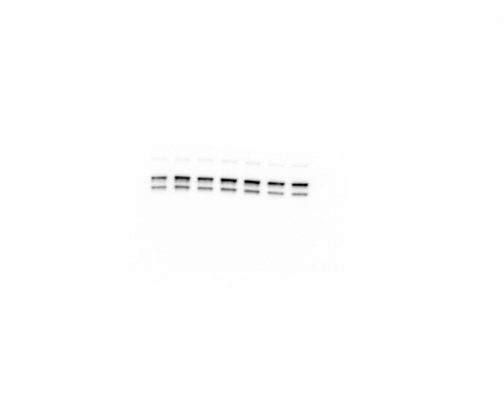


**β-actin**




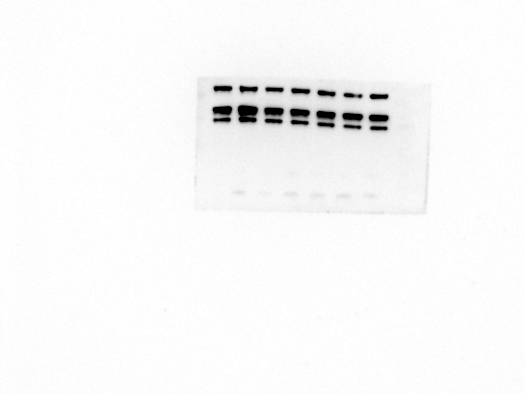


**Gels cut before and after**


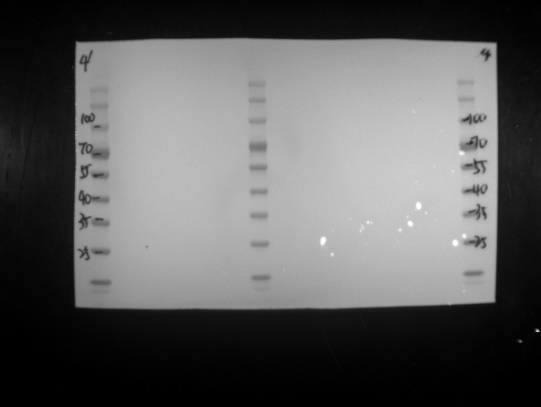

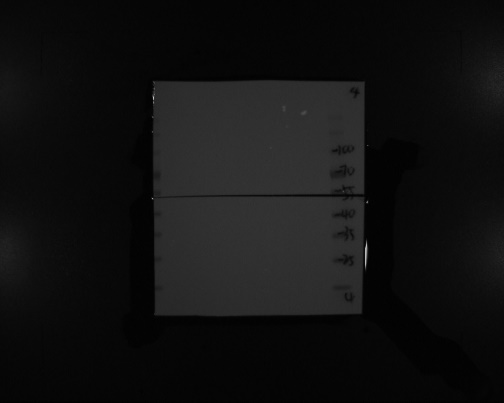


Figure6E

Sham/Saline/Niacin

**SQSTM1**


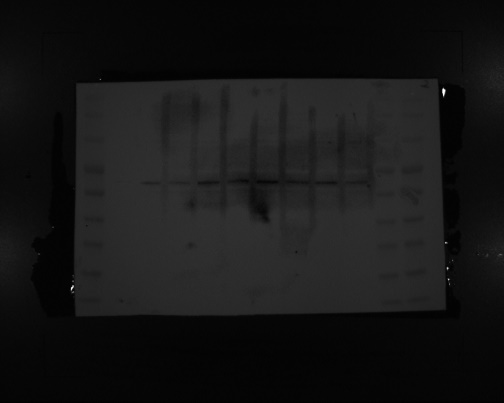

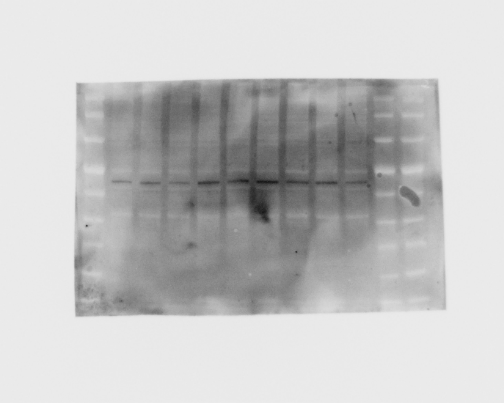


**β-actin**


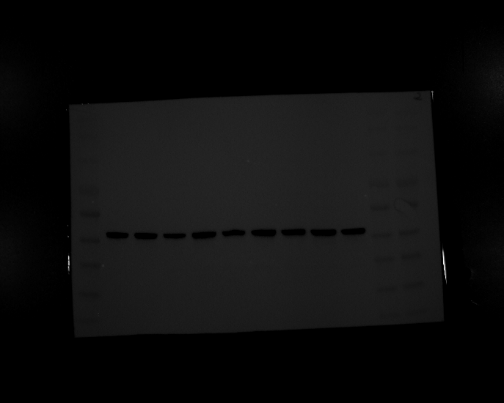

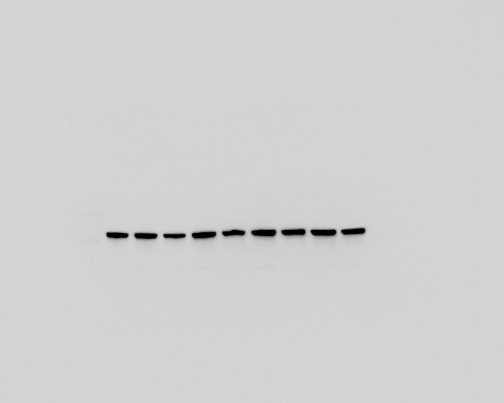


**Beclin1**


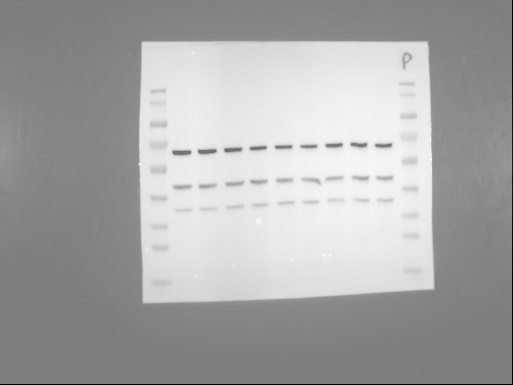

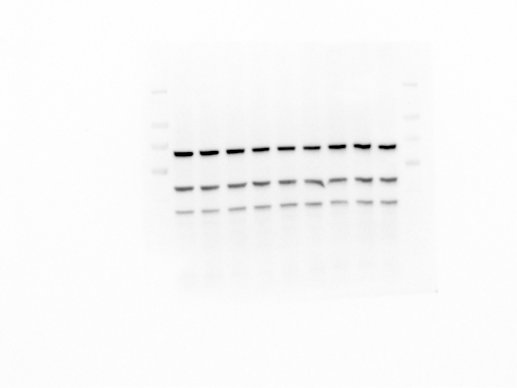


**β-actin**


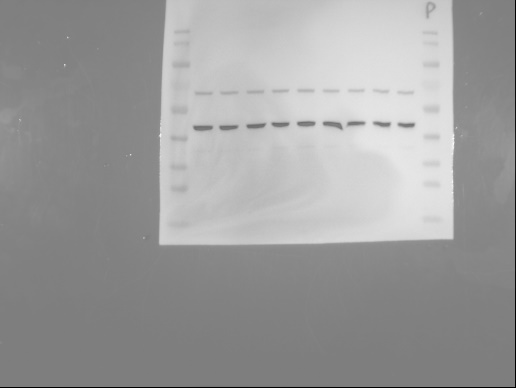

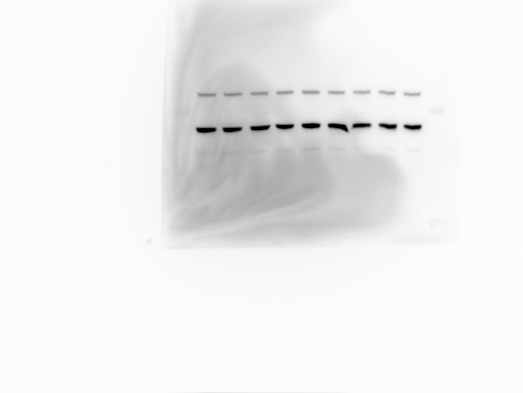


Figure6F

Sham/Saline/Niacin/Niacin+EX527/Niacin+ oss_128167

**Beclin1**


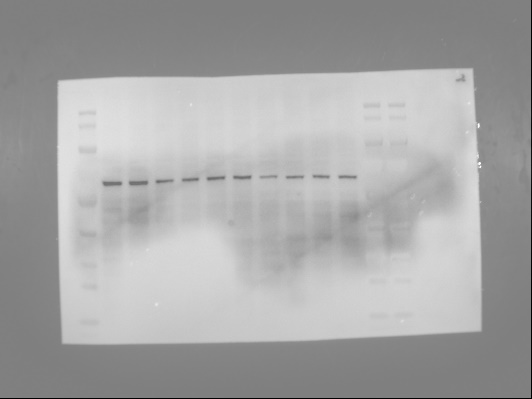

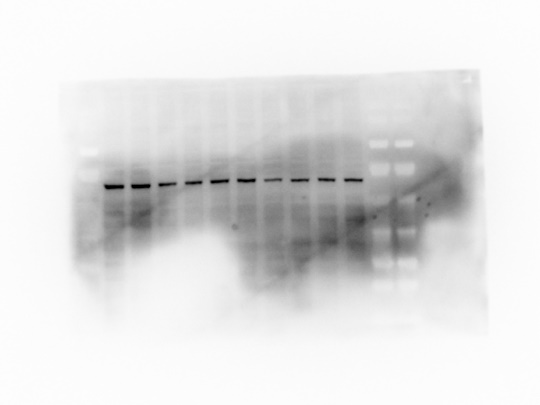


**GAPDH**


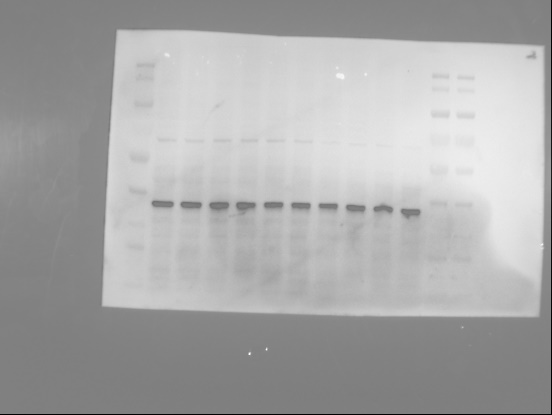

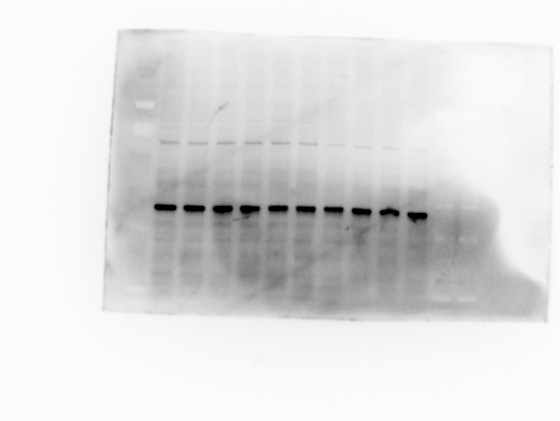


**QSQTM1**

**β-actin**

Supplementary FigureS1 E

Sham/Saline/Niacin (vascular)

**Runx2**

**OPN**

**GAPDH**

**Gels cut before and after**

Supplementary FigureS3

**Sirt1**

**β-actin**

**Sirt6**

**β-actin**
